# QTL‐seq approach identified genomic regions and diagnostic markers for rust and late leaf spot resistance in groundnut (*Arachis hypogaea* L.)

**DOI:** 10.1111/pbi.12686

**Published:** 2017-02-07

**Authors:** Manish K. Pandey, Aamir W. Khan, Vikas K. Singh, Manish K. Vishwakarma, Yaduru Shasidhar, Vinay Kumar, Vanika Garg, Ramesh S. Bhat, Annapurna Chitikineni, Pasupuleti Janila, Baozhu Guo, Rajeev K. Varshney

**Affiliations:** ^1^ International Crops Research Institute for the Semi‐Arid Tropics (ICRISAT) Hyderabad India; ^2^ Department of Biotechnology University of Agricultural Sciences Dharwad India; ^3^ Crop Protection and Management Research Unit USDA‐Agricultural Research Service Tifton GA USA; ^4^ School of Plant Biology and Institute of Agriculture The University of Western Australia Crawley WA Australia

**Keywords:** resequencing, trait mapping, QTL‐seq analysis, candidate gene discovery, diagnostic markers

## Abstract

Rust and late leaf spot (LLS) are the two major foliar fungal diseases in groundnut, and their co‐occurrence leads to significant yield loss in addition to the deterioration of fodder quality. To identify candidate genomic regions controlling resistance to rust and LLS, whole‐genome resequencing (WGRS)‐based approach referred as ‘QTL‐seq’ was deployed. A total of 231.67 Gb raw and 192.10 Gb of clean sequence data were generated through WGRS of resistant parent and the resistant and susceptible bulks for rust and LLS. Sequence analysis of bulks for rust and LLS with reference‐guided resistant parent assembly identified 3136 single‐nucleotide polymorphisms (SNPs) for rust and 66 SNPs for LLS with the read depth of ≥7 in the identified genomic region on pseudomolecule A03. Detailed analysis identified 30 nonsynonymous SNPs affecting 25 candidate genes for rust resistance, while 14 intronic and three synonymous SNPs affecting nine candidate genes for LLS resistance. Subsequently, allele‐specific diagnostic markers were identified for three SNPs for rust resistance and one SNP for LLS resistance. Genotyping of one RIL population (TAG 24 × GPBD 4) with these four diagnostic markers revealed higher phenotypic variation for these two diseases. These results suggest usefulness of QTL‐seq approach in precise and rapid identification of candidate genomic regions and development of diagnostic markers for breeding applications.

## Introduction

Groundnut or peanut (*Arachis hypogaea* L.) is one of the major sources of vegetable oil (48%) and protein (25%) in the semi‐arid tropics. This crop is grown in more than 100 countries worldwide with the total production of 42.4 million tons from 25.7 million ha area during 2014 (http://faostat.fao.org/). Two foliar fungal diseases namely rust (caused by *Puccinia arachidis*) and late leaf spot (LLS) (caused by *Cercosporidium personatum*) cause severe yield loss and reduce fodder quality. When both diseases occur simultaneously, the damage could lead to 50%–70% yield loss (Subramanyam *et al*., 1984). For instance in an estimate in 2009, a loss of $326 million by early leaf spot, $467 million by rust and $599 million by LLS was estimated (Monyo *et al*., 2009). Although fungicides are available to control these diseases, their application increases financial burden on farmers, thereby increasing the production cost and reduction in the marginal income. The application of fungicides also has detrimental effects on human health, soil, underground water and environment (Monyo *et al*., 2009). As the control measures using fungicides are neither cost‐effective nor environment‐friendly, breeding new cultivars with genetic resistance is sustainable and environment‐friendly approach.

With the lower productivity and increasing demand supply, the goal is to develop high‐yielding varieties equipped with resistance/tolerance to biotic and abiotic stresses. The conventional breeding alone may not be able to achieve above required milestone and the integration of genomics tools with the conventional breeding approaches would be the best option to achieve accelerated genetic gains through genomics‐assisted breeding (GAB) (Pandey *et al*., 2012; Varshney *et al*., 2013; Varshney 2015). However, availability of linked markers to the trait of interest is prerequisite to deploy the most successful GAB approach, such as marker‐assisted backcrossing (MABC). The identification of user‐friendly markers for these foliar fungal diseases is required to improve resistance against rust and LLS diseases in groundnut. The earlier studies identified one major quantitative trait locus (QTL) for rust and two major QTLs for LLS resistance using the recombinant inbred line (RIL) population derived from the cross TAG 24 × GPBD 4 (Khedikar *et al*., 2010; Sujay *et al*., 2012). These studies provided linked markers for rust and LLS resistance. The QTL for rust resistance showed 82.6% phenotypic variance explained (PVE), while both the QTLs for LLS resistance showed 40%–60% PVE. The linked simple sequence repeat (SSR) markers identified from these studies were validated and deployed through MABC to improve resistance for rust and LLS in three elite varieties (Varshney *et al*., 2014a). The linked marker, IPAHM103, for rust resistance identified by Khedikar *et al*. (2010) and Sujay *et al*. (2012) in TAG 24 × GPBD 4 and TG 26 × GPBD 4 mapping populations was also detected by Mondal *et al*. (2012) in the VG 9514 × TAG 24 mapping population indicating the same genomic segment conferring rust resistance that has come from the same accession ICGV 86855 of *Arachis cardenasii* in both resistant genotypes (GPBD 4 and VG9514).

Draft genome sequences for both the diploid progenitors of tetraploid cultivated groundnut have become available recently (Bertioli *et al*., 2016; Chen *et al*., 2016) that could help in finding the genes and SNPs present in the QTL regions on the diploid genomes. It is important to note that one major QTL each for both diseases was colocalized on linkage group AhXV (now A03), after genome sequencing and assigning the pseudomolecules, Bertioli *et al*. (2016), while the second major QTL for LLS resistance was located on linkage group AhXII (now A02). It is technically difficult to genotype the populations with the currently available linked markers. Furthermore, unclear banding pattern when genotyped on polyacrylamide gel electrophoresis (PAGE) and complicated peak pattern when analysed on the capillary electrophoresis demands repetition of experiments. The other issue is timing involved in genotyping the segregating breeding populations to select the true hybrid F_1_ plants for making backcrosses, which gives only 8–10 days of time window before flowering ends. The above technical issues hindered large‐scale adoption and deployment of these linked markers in small‐to‐medium‐sized genotyping laboratories in developing countries. Therefore, it would be appropriate to dissect these QTLs in order to identify candidate genes controlling the resistance to rust and LLS and to develop user‐friendly diagnostic markers for use in GAB.

The evolution in the next‐generation sequencing technologies (NGS) in the last decade has drastically reduced cost of sequencing that has enabled use of sequence‐based trait mapping approaches to identify the markers (Varshney *et al*., 2014b). As compared to traditional QTL mapping approach using RIL population, the sequence‐based trait mapping through generation of whole‐genome resequencing (WGRS) data on complete or partial mapping population facilitates identification of genomewide large number of single‐nucleotide polymorphisms (SNPs) and more specifically from the target candidate QTL region controlling traits of interest (Chen *et al*., 2014; Pandey *et al*., 2016; Qi *et al*., 2014; Xu *et al*., 2013). In case of simple traits under oligogenic control such as rust and LLS resistance in groundnut, the cost can be further reduced using bulk segregant analysis (BSA) to identify the markers linked to the trait of interest (Michelmore *et al*., 1991)_._ The BSA can be more effectively deployed using the NGS technology by generating sequence data on the extreme bulks and parental genotypes, popularly known as QTL‐seq approach, to locate the candidate genomic regions and underlying genes more rapidly (Takagi *et al*., 2013). This approach has been successfully deployed in locating the genomic regions and identifying candidate genes in several crops such as cucumber (Lu *et al*., 2014)_,_ tomato (Illa‐Berenguer *et al*., 2015), pigeonpea (Singh *et al*., 2016a) and chickpea (Das *et al*., 2015; Singh *et al*., 2016). Therefore, this approach was deployed to locate the genomic region and candidate genes associated with resistance to rust and LLS in groundnut.

## Results

### Phenotypic diversity in RIL population and construction of bulks

**Figure 1:**
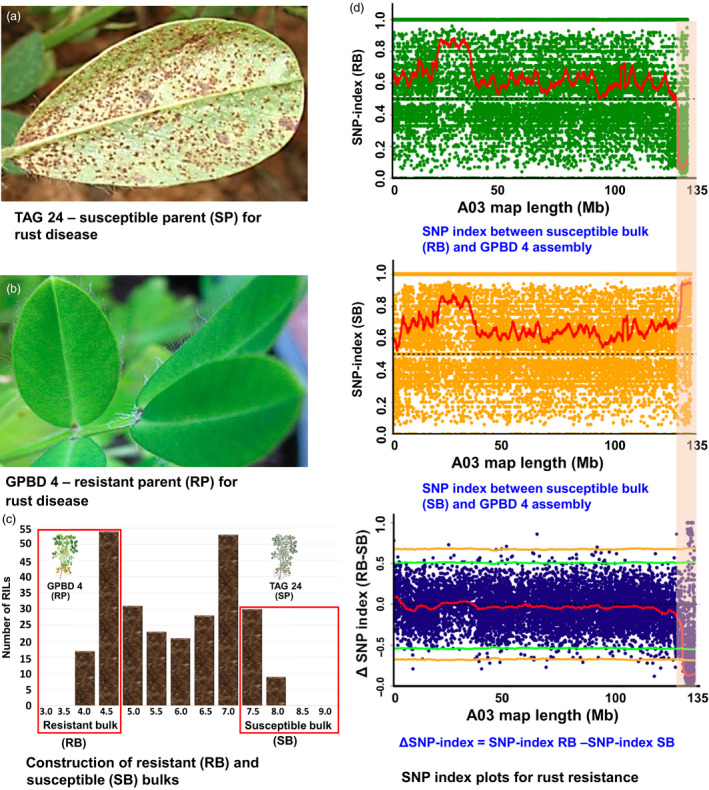
QTL‐seq approach for mapping genomic regions controlling rust resistance. (a) TAG 24: susceptible parent for rust disease; (b) GPBD 4: resistant parent for rust disease; (c) frequency distribution for rust resistance showing phenotypic variation in RIL population. The DNA of 25 RILs with extreme phenotypes (high and low disease score) was used to develop susceptible and resistant bulks; (d) SNP index plot between resistant bulk and GPBD 4 assembly (top), susceptible and GPBD 4 assembly (middle) and ∆SNP index plot (bottom) of pseudomolecule A03 with statistical confidence interval under the null hypothesis of no QTLs (orange, *P* < 0.01 and green *P* < 0.05). The significant genomic region identified for rust resistance is shaded (131.60–134.66 Mb).

**Figure 2:**
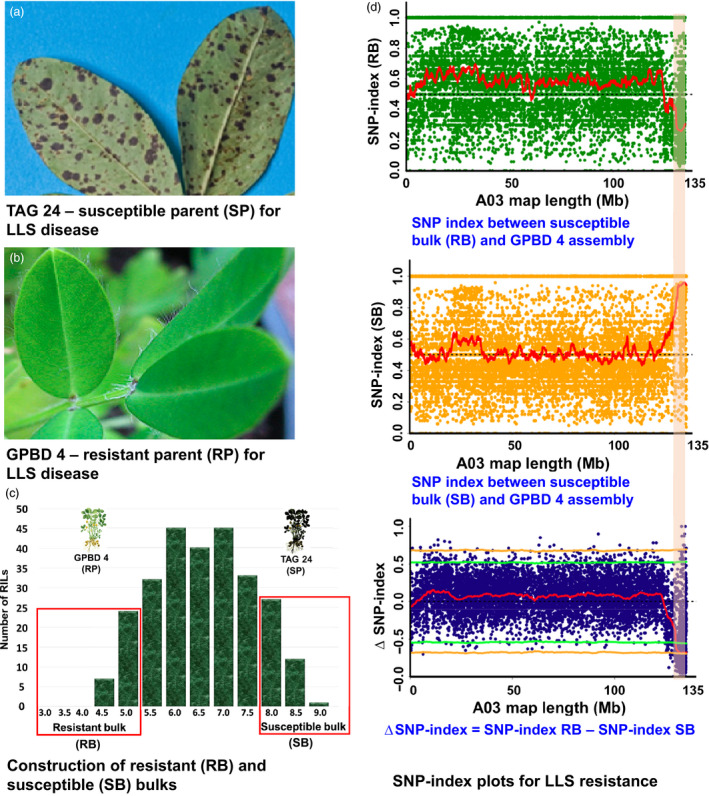
QTL‐seq approach for mapping genomic regions controlling late leaf spot resistance. (a) TAG 24: Susceptible parent for LLS disease; (b) GPBD 4: Resistant parent for LLS disease; (c) Frequency distribution for LLS resistance showing phenotypic variation in RIL population. The DNA of 25 RILs with extreme phenotypes (high and low disease score) was used to develop susceptible and resistant bulks; (d) SNP index plot between resistant bulk and GPBD 4 assembly (top), susceptible and GPBD 4 assembly (middle) and ∆SNP index plot (bottom) of pseudomolecule A03 with statistical confidence interval under the null hypothesis of no QTLs (orange, *P* < 0.01 and green *P* < 0.05). The significant genomic region identified for LLS resistance is shaded (131.67–134.65 Mb).

The RIL population (TAG 24 × GPBD 4) used in this study had high phenotypic variability for both diseases, rust and LLS (Figures 1 and 2). Therefore, resistant and susceptible bulks were constituted by mixing equimolar DNA from 25 RILs with extreme phenotypes, that is resistant and susceptible for both the diseases as shown in Figures S1 and S2. In the RIL population, the disease score for rust disease ranged from 3.4 (RIL‐146) to 8.1 (RIL‐166), while for LLS, it varied from 3.5 (RIL‐2) to 8.5 (RIL‐216) (Table S1). The average disease score for rust disease was 3.7 for resistant bulk and 7.7 for susceptible bulk, while the average disease score for LLS disease was 4.4 for resistant bulk and 8.1 for susceptible bulk. The mean disease score for susceptible (TAG 24) parent for rust and LLS disease was 7.5 and 8.4, respectively, while the mean disease score of resistant parent (GPBD 4) for rust and LLS resistance was 3.0 and 3.7, respectively. The Figure S1 shows the phenotypic variability in the RIL population and between susceptible as well as resistant bulks.

### Sequencing and mapping of reads to the genome

The WGRS data were generated for five samples namely GPBD 4 (resistant parent for rust and LLS), resistant bulk for rust (Rust_Rbulk), susceptible bulk for rust (Rust_Sbulk), resistant bulk for LLS (LLS_Rbulk) and susceptible bulk for LLS (LLS_Sbulk). A total of 395.70 million reads for resistant parent (GPBD 4), 423.76 million reads for (Rust_Rbulk), 371.52 million reads for (Rust_Sbulk), 365.22 million reads for (LLS_Rbulk) and 384.24 million reads for (LLS_Sbulk) were generated (Tables 1 and S2). The maximum sequencing data were obtained for Rust_Rbulk (41.95 Gb) followed by resistant parent (39.17 Gb), LLS_Sbulk (38.04 Gb), Rust_Sbulk (36.78 Gb) and LLS_Rbulk (36.16 Gb). The highest mapping of reads to the genome was obtained for the resistant parent (280.77 million reads) followed by Rust_Rbulk (270.88 million reads), Rust_Sbulk (266.82 million reads), LLS_Sbulk (249.85 million reads) and LLS_Rbulk (249.60 million reads).

**Table 1:** Summary of disease score and Illumina sequencing of parental lines and bulks for rust and late leaf spot resistance

Sample	Mean disease score		Illumina sequencing			
	Rust	LLS	Data generated (Gb)	% Alignment	% Genome coverage	Average depth (X)
GPBD 4^a^	3.0	3.7	39.17	95.8	86.6	11.6
Rust_Rbulk^b^	3.7		41.95	95.8	86.8	11.2
Rust_Sbulk^b^	7.7		36.78	94.4	86.9	11.0
LLS_Rbulk^b^		4.4	36.16	96.5	86.6	10.3
LLS_Sbulk^b^		8.1	38.04	96.5	86.6	10.3

a GPBD 4 short reads were aligned to the publicly available genome of diploid progenitors *Arachis duranensis* and *Arachis ipaensis* (PeanutBase: http://peanutbase.org/).

b The short reads of bulks were aligned to the GPBD 4 ‘reference sequence’ developed by replacement of SNPs between GPBD 4 and diploid progenitors.

The alignment of reads generated for the resistant genotype (GPBD 4) achieved 86.57% genome coverage and 11.6 X of average read depth and resulted in development of reference‐guided based assembly, that is GPBD 4 assembly (Figure S2). In the case of rust resistance, mapping of reads for Rust_Rbulk to the GPBD 4 assembly resulted in 86.75% coverage and 11.2 X read depth, while Rust_Sbulk to the GPBD 4 assembly resulted in 86.86% coverage and 11.0 X read depth (Tables 1 and S2). Similarly for LLS resistance, mapping of reads for LLS_Rbulk to the GPBD 4 assembly resulted in 86.64% coverage and 10.3 X read depth, while LLS_Sbulk to the GPBD 4 assembly resulted in 86.62% coverage and 10.3 X read depth. After analysing the resistant and susceptible bulks, a total of 259 621 genomewide SNPs for rust resistance, while 243 262 genomewide SNPs for LLS were identified (Table S3). Of these, 75 203 SNPs for rust and 62 358 SNPs for LLS were homozygous between bulks which were used for further investigation and identification of effective SNPs.

### Candidate genomic region(s) for rust and late leaf spot resistance

To identify the candidate genomic region(s) controlling resistance to rust and LLS, the SNP index was calculated for each bulk by comparing to the GPBD 4 assembly. In simple terms, the frequency of parental alleles in the population of bulked samples represents the SNP index. For example, the SNP index will be 0.5 if both the parents contribute equally to the population. The deviation of allele frequency from 0.5 indicates presence of more alleles of one parent than the other for a particular genomic position. Therefore, genomewide SNP index was calculated with the sliding window of 2‐Mb interval with 50 kb increment for resistant and susceptible bulks to detect the candidate genomic regions which deviated from 0.5 for both the diseases (Figures [Link], [Link], [Link], [Link], [Link], [Link], [Link]). After calculating the SNP index, ∆SNP index with a statistical confidence of *P* < 0.05, significant genomic positions were identified on A03 linkage group for both the disease.

For rust resistance, 3.06 Mb (131.60–134.66 Mb) genomic region was identified after analysing the sequences of resistant and susceptible bulk on the A03 pseudomolecule of A‐genome (Figure 1). This genomic region had 3136 SNPs with read depth of ≥7 and ∆SNP index = −1. The negative sign of ∆SNP index indicates presence of biasedness in the inheritance of parental genomes in the bulks towards resistant parent (Table S3). The resistant bulk had SNP index = 0 at all the 3136 SNP positions indicating the contribution of alleles coming from the resistant parent GPBD 4 (Table S4). Similarly, the susceptible bulk scored SNP index = 1 indicating the source of alleles for susceptibility from susceptible parent TAG 24. Of the 3136 SNPs, 2455 SNPs were intergenic, 434 intronic, 30 nonsynonymous, one resulted in stop codon, 144 synonymous, two without any effect, 58 in 3′ UTR and 12 in 5′ UTR. The above approach identified 30 nonsynonymous SNPs affected 25 candidate genes relating to plant growth and defence (Table 2).

**Table 2:** Identification of SNPs in putative candidate genes in the genomic region for rust resistance on pseudomolecule A03

Gene	Position (bp)	GPBD 4 assembly (resistant parent) base	Resistant bulk base	Susceptible bulk base	ΔSNP index	Amino acid change	Function	U99	L99
Nonsynonymous SNPs and candidate genes for rust resistance									
*Aradu.FAV4Y*	131657367	C	C	A	−1	caG/caT	2‐oxoglutarate (2OG) and Fe(II)‐dependent oxygenase superfamily protein	0.714	−0.714
	131657379	G	G	C	−1	caC/caG		0.700	−0.700
*Aradu.H1HIG*	131739517	A	A	C	−1	Tct/Gct	Purple acid phosphatase	0.700	−0.700
*Aradu.L0AQP*	131752809	T	T	C	−1	gAa/gGa	Unknown protein	0.700	−0.700
*Aradu.7MV8U*	131783499	C	C	G	−1	Gtg/Ctg	Transthyretin‐like protein	0.714	−0.714
	131783520	C	C	T	−1	Gat/Aat		0.750	−0.750
*Aradu.PNQ8T*	131788843	C	C	T	−1	Gaa/Aaa	Unknown protein	0.714	−0.714
*Aradu.9C8P4*	131918196	G	G	C	−1	cCa/cGa	Protein kinase superfamily protein	0.636	−0.636
*Aradu.14X1M*	131937796	G	G	A	−1	Gat/Aat	ATP binding microtubule motor family protein isoform 1	0.750	−0.750
	131938803	A	A	T	−1	aAc/aTc		0.615	−0.615
*Aradu.N7C0U*	131950239	A	A	T	−1	Atg/Ttg	Reticulon family protein	0.750	−0.750
*Aradu.AB2YQ*	132022031	C	C	T	−1	Cat/Tat	C2H2‐like zinc finger protein	0.750	−0.750
*Aradu.5N8I2*	132617185	C	C	T	−1	gCg/gTg	Remorin‐like	0.714	−0.714
*Aradu.7P7FQ*	132700619	A	A	G	−1	Aca/Gca	Alpha/beta‐Hydrolases superfamily protein	0.750	−0.750
*Aradu.1ZB11*	132977576	C	C	T	−1	tCg/tTg	Dentin sialophosphoprotein‐like isoform X4	0.700	−0.700
*Aradu.B0A4N*	133407585	C	C	T	−1	Gag/Aag	ATP binding microtubule motor family protein	0.714	−0.714
*Aradu.6U7NW*	133497786	T	T	A	−1	Agc/Tgc	Uncharacterized protein	0.643	−0.643
	133498045	G	G	T	−1	ttC/ttA		0.636	−0.636
*Aradu.KU7EH*	133527661	C	C	G	−1	caC/caG	UDP‐Glycosyltransferase superfamily protein	0.714	−0.714
*Aradu.3AT2D*	133594028	A	A	C	−1	Agc/Cgc	Selenium‐binding protein	0.750	−0.750
*Aradu.Z87JB*	133780314	T	T	C	−1	Att/Gtt	Disease resistance protein (TIR‐NBS‐LRR class)	0.750	−0.750
*Aradu.L63AM*	133783696	G	G	A	−1	Gat/Aat	PIF1‐like helicase	0.700	−0.700
*Aradu.9E85R*	133796773	G	G	A	−1	aGa/aAa	Beta galactosidase	0.750	−0.750
*Aradu.N20HG*	133814877	G	G	T	−1	aCt/aAt	ATP/DNA‐binding protein	0.750	−0.750
*Aradu.NG5IQ*	133999438	G	G	C	−1	tCt/tGt	Glucan endo‐1%2C3‐beta‐glucosidase 4‐like	0.714	−0.714
*Aradu.G696X*	134170720	C	C	T	−1	Cgt/Tgt	Alpha/beta‐hydrolase superfamily protein	0.643	−0.643
*Aradu.H715D*	134280699	G	G	A	−1	Cgc/Agc	Uncharacterized protein	0.667	−0.667
	134280707	G	G	C	−1	aaG/aaC	Isoform X4	0.750	−0.750
*Aradu.YAN03*	134343833	A	A	C	−1	caA/caC	Nucleobase‐ascorbate transporter	0.700	−0.700
*Aradu.LSV4Q*	134476055	T	T	C	−1	Atc/Gtc	NADH: ubiquinone oxidoreductase intermediate‐associated protein	0.750	−0.750

ΔSNP index of each SNP positions was calculated using following formula: ΔSNP index = SNP index of susceptible bulk—SNP index of resistant bulk. U99: 99% confidence interval upper side; L99: 99% confidence interval lower side.

**Figure 3:**
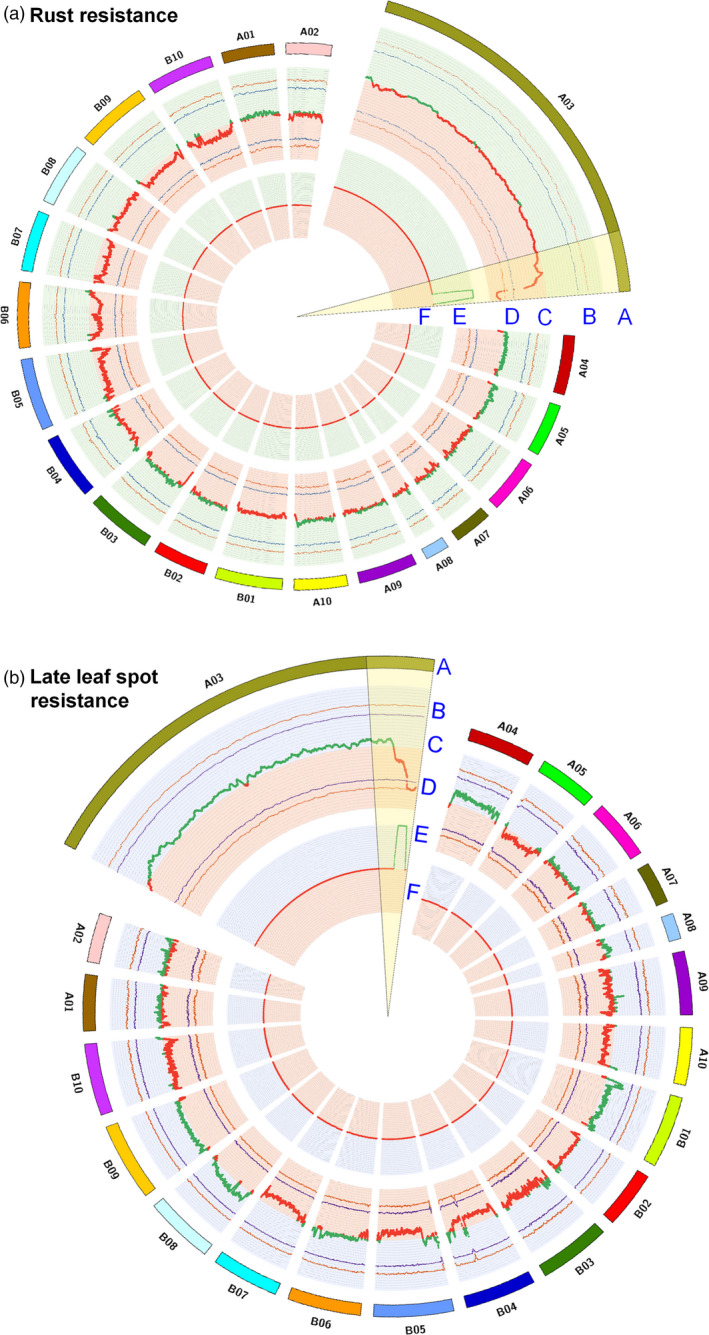
Colocalization of QTLs identified through traditional genetic mapping and QTL‐seq approach for resistance to rust and late leaf spot. (a) Colocalization of QTLs mapped for rust resistance through traditional and QTL‐seq method. (A) Psuedomolecules of reference genome *Arachis duranensis* (B) Upper probability values at 99% confidence (*P* < 0.01) and 95% confidence (*P* < 0.05) for declaring significant ΔSNP index (C) genomewide ΔSNP index (red dots denote ΔSNP index ranged from 0 to −1 and contributed by susceptible parent (TAG 24) and green dots denote ΔSNP index ranged from 0 to 1 and contributed by resistant parent (GPBD 4), (D) lower probability values at 99% confidence (*P* < 0.01) and 95% confidence (*P* < 0.05), (E) physical position of earlier mapped QTL (Sujay *et al*., 2012) for rust resistance through traditional mapping approach. The physical position of QTL was estimated through blast the flanking primers into the *A. duranensis* genome and (F) common genomic positions on pseudomolecule A03 were observed through both the approaches. (b) Colocalization of QTLs mapped for LLS resistance through traditional and QTL‐seq method. (A) Psuedomolecules of reference genome *A. duranensis,* (B) Upper probability values at 99% confidence (*P* < 0.01) and 95% confidence (*P* < 0.05) for declaring significant ΔSNP index, (C) genomewide ΔSNP index (red dots denote ΔSNP index ranged from 0 to −1 and contributed by susceptible parent (TAG 24) and green dots denote ΔSNP index ranged from 0 to 1 and contributed by resistant parent (GPBD 4), (D) lower probability values at 99% confidence (*P* < 0.01) and 95% confidence (*P* < 0.05), (E) physical position of earlier mapped QTL (Sujay *et al*., 2012) for late leaf spot resistance through traditional mapping approach. The physical position of QTL was estimated through blast the flanking primers into the *A. duranensis* genome, and (F) common genomic positions on pseudomolecule A03 were observed through both the approaches.

Similarly for LLS resistance, 2.98 Mb (131.67–134.65 Mb) genomic region was identified upon analysing the sequences of resistant and susceptible bulk on A03 pseudomolecule (Figure 2). This is the same genomic region as detected for rust resistance as detected above for rust resistance. This genomic region contained 66 SNPs with a minimum read depth of 7 and ∆SNP index = −1 (Table S5). The resistant bulk had SNP index = 0 at all 66 SNP positions indicating the contribution of alleles coming from the resistant parent GPBD 4, while the susceptible bulk scored SNP index = 1 indicating the source of susceptibility alleles from susceptible parent TAG 24. Of the 66 SNPs, no SNP was nonsynonymous. However, 14 intronic and three synonymous SNPs were identified in nine candidate genes (Table 3). Further, the genomic region identified for rust and LLS resistance on pseudomolecule A03 were overlapped. Interestingly, the genomic region is underlying the QTL identified earlier by traditional QTL mapping (Sujay *et al*. 2012), for rust and LLS resistance (Figure 3).

**Table 3:** Identification of SNPs in putative candidate genes in the identified genomic region on pseudomolecule A03 for late leaf spot resistance

Gene	Position (bp)	GPBD 4 assembly (resistant parent) base	Resistant bulk base	Susceptible bulk base	ΔSNP index	Amino acid change	Function	U99	L99
Intronic SNPs and candidate genes for LLS resistance									
*Aradu.PHU5I*	131755141	G	G	A	−1		Purple acid phosphatase 3	0.714	−0.714
	131755149	G	G	C	−1			0.750	−0.750
*Aradu.7MV8U*	131784975	G	G	A	−1		Transthyretin‐like protein	0.714	−0.714
	131784990	G	G	C	−1			0.667	−0.667
	131785313	T	T	C	−1			0.750	−0.750
	131785314	C	C	A	−1			0.750	−0.750
	131785428	G	G	A	−1			0.714	−0.714
*Aradu.RT35T*	131813401	T	T	C	−1		Xyloglucan endotransglucosylase/hydrolase	0.714	−0.714
*Aradu.RVF1V*	134565541	C	C	T	−1		Heat shock transcription factor	0.667	−0.667
*Aradu.98U3Z*	134642651	A	A	G	−1		Receptor kinase	0.667	−0.667
	134643689	C	C	T	−1			0.750	−0.750
	134644076	C	C	T	−1			0.714	−0.714
*Aradu.BS3D3*	134654808	T	T	A	−1		Membrane attack complex component/perforin (MACPF) domain protein	0.750	−0.750
	134656184	C	C	A	−1			0.714	−0.714
Synonymous SNPs and candidate genes for LLS resistance									
*Aradu.8X6B9*	131844849	G	G	A	−1	Att	Cytochrome	0.750	−0.750
*Aradu.VP5WD*	134284373	G	C	A	−1	aaT	Putative Myb family transcription factor	0.667	−0.667
*Aradu.V4NFM*	134503983	C		T	−1	ctT	Glutathione S‐transferase family protein	0.714	−0.714

ΔSNP index of each SNP positions was calculated using following formula: ΔSNP index = SNP index of susceptible bulk—SNP index of resistant bulk. U99: 99% confidence interval upper side; L99: 99% confidence interval lower side.

### Putative candidate genes associated with rust and late leaf spot resistance

Of the 25 putative candidate genes found associated with rust resistance, four putative candidate genes (*Aradu.L0AQP, Aradu.PNQ8T, Aradu.6U7NW* and *Aradu.H715D*) were predicted to code for either uncharacterized or unknown protein (Table 2). Two putative candidate genes namely *Aradu.7P7FQ* and *Aradu.G696X* code for alpha/beta‐hydrolase superfamily protein. The remaining putative candidate genes code for different types of proteins such as ATP binding microtubule motor family (*Aradu.B0A4N*), ATP/DNA‐binding (*Aradu.N20HG*), 2‐oxoglutarate (2OG) and Fe(II)‐dependent oxygenase superfamily (*Aradu.FAV4Y*), purple acid phosphatase (*Aradu.H1HIG*), transthyretin‐like (*Aradu.7MV8U*), protein kinase superfamily (*Aradu.9C8P4*), reticulon family (*Aradu.N7C0U*), C2H2‐like zinc finger (*Aradu.AB2YQ*), remorin‐like (*Aradu.5N8I2*), dentin sialophosphoprotein‐like isoform (*Aradu.1ZB11*), UDP‐Glycosyltransferase superfamily (*Aradu.KU7EH*), disease resistance (TIR‐NBS‐LRR class) (*Aradu.Z87JB*), PIF1‐like helicase (*Aradu.L63AM*), beta galactosidase (*Aradu.9E85R*), glucan endo‐1%2C3‐beta‐glucosidase4‐like (*Aradu.NG5IQ*), NADH:ubiquinone oxidoreductase intermediate‐associated (*Aradu.LSV4Q*) and nucleobase‐ascorbate transporter (*Aradu.YAN03*).

Similarly for LLS resistance, total nine putative candidate genes were identified which code for different types of proteins such as purple acid phosphatase (*Aradu.PHU5I*), transthyretin‐like protein (*Aradu.7MV8U*), xyloglucan endotransglucosylase/hydrolase (*Aradu.RT35T*), heat shock transcription factor (*Aradu.RVF1V*), receptor kinase (*Aradu.98U3Z*), MACPF domain (*Aradu.BS3D3*), cytochrome B561 (*Aradu.8X6B9*) and putative Myb family transcription factor (*Aradu.VP5WD*) and glutathione S‐transferase family (*Aradu.V4NFM*) (Table 3). A maximum of five effective SNPs were identified for putative candidate gene *Aradu.7MV8U*.

### Marker development, genetic map and QTL analysis

A total of 47 SNPs (30 SNPs for rust and 17 SNPs for LLS resistance) were targeted for development of allele‐specific markers. Of the 30 SNPs for rust resistance, allele‐specific primers were successfully developed for 17 SNPs, while no primers could be designed for remaining 13 SNPs. Of the 17 SNPs for rust resistance, primers were developed for both alleles of 14 SNPs and single allele of remaining three SNPs. Similarly for LLS resistance, 17 SNPs were targeted for primer designing. Of the 17 SNPs, primers were successfully developed for eight SNPs, while no primer was designed for remaining nine SNPs. Of the eight SNPs for LLS, primers were developed for both alleles of six SNPs and single allele of remaining two SNPs. In total, a total of 45 allele‐specific markers were developed for potential use in breeding, that is 31 for rust resistance and 14 for LLS resistance (Table S6).

All 45 allele‐specific markers were checked for polymorphism between parental genotypes of the RIL population (TAG 24 × GPBD 4). Of the 45 markers, 36 markers (27 for rust and nine for LLS resistance) gave good amplification, while nine markers did not amplify in parental genotypes. Of the 36 amplified markers, only three (GMRQ517, GMRQ786 and GMRQ843) markers for rust resistance and one (GMLQ975) marker for LLS resistance were found polymorphic between parental genotypes. Of these four markers, three markers amplified the allele of resistant parent ‘GPBD 4’, while marker ‘GMRQ843’ amplified the allele of susceptible parent ‘TAG 24’. Complementary alleles of these markers were found monomorphic between the resistant and susceptible parents.

Genotyping data on complete mapping population were generated for these four polymorphic markers (three for rust resistance and one for LLS resistance) and were used for mapping to the linkage group (LG) of existing genetic map. All the four markers were mapped on the upstream of marker loci GM2009. The map distance of LG reduced from 116.5 cM to 94.4 cM, while marker loci increased from 12 to 16. QTL analysis using the genotyping and phenotyping data resulted in identification of one consistent QTL identified in different seasons between the marker loci GMRQ5157 and GM1536. The LOD value ranged from 3.5 to 49.9, while PVE varied from 9.0% to 83.6% (Table 4; Figure S11). This consistent QTL for rust resistance with 42.7–83.6% PVE identified in eight seasons while consistent QTL for LLS resistance with 9.0–63.1% PVE was identified in three seasons.

**Table 4:** Mapping of validated markers and re‐estimation of phenotypic effect for QTLs controlling rust and late leaf spot resistance

QTLs	Position (cM)	LOD value	Marker interval	Nearest marker	Phenotypic variance explained (PVE%)	Additive effect (a0)
Rust resistance						
*qRust80D_06*	31.6	36.1	GMRQ517‐Seq2B10	IPAHM103	83.6	1.365
*qRust90D_06*	30.6	24.1	GMRQ517‐Seq2B10	IPAHM103	75.4	1.540
*qRust 80D_07*	31.6	49.9	GMRQ517‐Seq2B10	IPAHM103	65.4	1.307
*qRust 90D_07*	31.6	47.2	GMRQ517‐Seq2B10	IPAHM103	73.1	1.309
*qRust 80D_08*	31.6	35.2	GMRQ843‐Seq2B10	IPAHM103	69.7	0.946
*qRust 90D_08*	31.6	49.2	GMRQ517‐Seq2B10	IPAHM103	63.7	1.977
*qRust 80D_09*	31.6	16.0	GMRQ517‐Seq2B10	IPAHM103	48.9	0.896
*qRust 90D_09*	31.6	14.6	GMRQ517‐Seq2B10	IPAHM103	42.7	1.036
Late leaf spot resistance						
*qLLS70D_08*	31.6	4.6	GM2009‐Seq2B10	IPAHM103	14.9	−0.279
*qLLS 90D_08*	30.6	21.1	GMRQ517‐Seq2B10	IPAHM103	63.1	−1.415
*qLLS 90D_09*	26.2	3.5	GMRQ517‐Seq2B10	GM2009	9.0	−0.492

GMRQ517 and GMRQ843 are the newly designed markers from this study.

### Validation of allele‐specific markers

A total of 45 allele‐specific markers developed in this study were used for validation and identification of diagnostic markers for these two foliar diseases. Although the initial screening on parental genotypes of the RIL population produced amplification for 36 markers, only five of these markers could be scored for polymorphic alleles. These five polymorphic markers were then validated on a panel of diverse genotypes containing susceptible genotypes (GJ 9, GJ 20, GJGHPS 1, SunOleic 95R, ICGV 07368, ICGV 06420, TMV 2, DH 86, TAG 24, TG 26, ICGV 91114 and JL 24), resistant parent (GPBD 4) of the RIL population and 11 introgression lines (four in the genetic background of ICGV 91114, three in JL 24 and four in TAG 24) developed through marker‐assisted backcrossing (MABC) approach. Of these five markers, three markers (GMRQ517, GMRQ786 and GMRQ843) showed clear differentiation between resistant and susceptible genotypes for rust resistance, while one marker (GMLQ975) was identified for LLS resistance (Table 5; Figure S12). The first diagnostic marker ‘GMRQ517’ for rust resistance amplified 150‐bp fragment in the resistant parent and null allele in the susceptible genotypes. The second diagnostic marker for rust resistance ‘GMRQ786’ amplified 200‐bp fragment in the resistant parent and null allele in rust susceptible genotypes. In contrast to these two diagnostic markers, the third diagnostic marker ‘GMRQ843’ amplified 200‐bp fragment in the susceptible parent and null allele in resistant genotypes. Most importantly, these three diagnostic markers can be used in combination (GMRQ517 + GMRQ843) in the segregating population to differentiate the homozygotes and heterozygotes; that is, resistant lines will have 150‐bp allele from marker ‘GMRQ517’ and susceptible lines will have a 200‐bp allele from marker ‘GMRQ843’. In case of LLS resistance, the diagnostic marker ‘GMLQ975’ amplified a 150‐bp band in the resistant parent and null allele in susceptible genotypes. These markers are very useful for selecting breeding lines with resistance to rust and LLS.

**Table 5:** Validated user‐friendly diagnostic markers for rust and LLS resistance for use in genomics‐assisted breeding

Trait	Diagnostic markers	Forward sequence	Reverse sequence	Annealing temperature (°C)	Amplicon size (bp)
Rust	GMRQ517	TGTACCTGAAATGCAAGTTGAGAC	AATGTATGTGTGTTGGGCCC	59	150
Rust	GMRQ786	AACATTGTAACACTCACCTGGCTA	TCATGCTTGAACTGTGCCTC	59	200
Rust	GMRQ843	AGCCTTGCGACTAGGTTCAT	CATGGTGAGAGACGCGTAAG	59	200
LLS	GMLQ975	GGTATCATGATGAATTTTTAGAAGACTAGG	GAAATTTGGCTTTGGGTTCA	59	150

## Discussion

Genomics‐assisted breeding (GAB) is a powerful tool for accelerated improvement of elite cultivars for few important and selected traits (Varshney *et al*., 2013). To deploy GAB in routine breeding programme in a given crop, making available tightly linked markers for agronomically important traits is the key to track the favourable alleles of target genes in the breeding population (Pandey *et al*., 2016). Of the two available trait mapping approaches, that is linkage mapping and linkage disequilibrium (LD) or association mapping, the success rate for identifying the linked markers with high PVE was higher in case of linkage mapping as majority of the markers currently deployed in GAB have come from linkage mapping approach. The linkage mapping requires development of mapping population by crossing two contrasting genotypes with diverse phenotypes followed by their genotyping and phenotyping to conduct QTL analysis for identification of linked markers. Similar to other crops, this approach has also been very successful in identifying linked markers for target traits in groundnut for traits like resistance to rust and LLS (Pandey *et al*., 2012, 2016; Varshney *et al*., 2013). The utility of such diagnostic markers has fostered breeding programmes leading to development of improved breeding lines for foliar disease resistance and oil quality in groundnut (Janila *et al*., 2016; Varshney *et al*., 2014a).

It is important to note that genetic map with optimum density is required for effective QTL identification and development of diagnostic markers for target traits. Studies conducted over last 7 years in groundnut have shown a very low level of polymorphism between the parental genotypes of the mapping populations (Varshney *et al*., 2013). The low polymorphism led to development of sparse/less dense genetic maps for QTL analysis which not only failed to provide tightly linked markers but also could not provide any information on the candidate genes controlling the target traits. The genetic mapping in cultivated groundnut started just 7 years back, that is 2009 when the first SSR‐based genetic map with 135 marker loci was developed using RIL population (TAG 24 × ICGV 86031) (Varshney *et al*., 2009). This study could achieve 12% polymorphism (150 SSR loci) upon screening a total of 1145 SSR markers on the parental genotypes. It was even more difficult to add markers to this map further as after screening another set of 2070 SSRs on parents, only 3% (65 SSRs) were found polymorphic which led to development of improved genetic map with mere 191 marker loci (Ravi *et al*., 2011). Realizing the genome size of tetraploid genome, the sparse genetic maps are not good for conducting high‐resolution mapping in groundnut. Nevertheless, genotyping‐by‐sequencing (GBS) approach has good potential in developing dense genetic maps for conducting high‐resolution genetic mapping (Zhou *et al*., 2014). However, recent advances in NGS technologies and availability of the reference genomes for both diploid progenitors (A‐ and B‐genome) have opened new opportunities for conducting high‐resolution trait mapping and identifying candidate genes/diagnostic markers quickly.

Of the several NGS‐based trait dissection and gene discovery approaches, QTL‐seq approach has been popular because it can rapidly detect genomic region(s) controlling target trait and candidate genes underlying in that region (Pandey *et al*., 2016; Takagi *et al*., 2013). It is important to note that QTL‐seq approach takes clues from the very popular trait mapping approach ‘bulked segregant analysis (BSA)’ proposed by Michelmore *et al*. (1991) and hence does not require genotyping of large population. This approach is a cost‐effective and is very successful when applied on a RIL population where multiseason phenotyping data are available for selection of appropriate RILs for pooling and sequencing. This approach has been successfully deployed for mapping: (i) blast resistance in rice (Takagi *et al*., 2013), (ii) early flowering trait in cucumber (Lu *et al*., 2014), (iii) fruit weight and locule number loci in tomato (Illa‐Berenguer *et al*., 2015), (iv) 100 seed weight and root traits in chickpea (Das *et al*., 2015; Singh *et al*., 2016) and (v) fusarium wilt and sterility mosaic disease resistance in pigeonpea (Singh *et al*., 2016a). This approach not only provides candidate genes for further cloning experiments but most importantly provides a variety of diagnostic markers for use in breeding.

The earlier genetic mapping studies with sparsely dense genetic maps identified one major QTLs for rust resistance in the RIL population (TAG 24 × GPBD 4) (Khedikar *et al*., 2010). Addition of more markers onto this genetic map helped in identification of the major QTL for rust resistance explaining up to 82.96% PVE, while the major QTL for LLS resistance explained up to 67.98% PVE (Sujay *et al*., 2012). The identified SSR markers for rust were then validated not only on germplasm but also were validated in two other RIL populations involving synthetic genotypes as one parent in another study (Sukruth *et al*., 2015). Similarly, another study (Kolekar *et al*., 2016) further added adding 139 new SSR and transposable element (TE) markers and detected the same QTL as detected by Sujay *et al*. (2012). While improving the earlier map developed by Sujay *et al*. (2012), Kolekar *et al*. (2016) experienced changed position and order of the markers on the map. In addition, two new TE markers linked to rust resistance were identified and validated. A difference in markers order among genetic maps was expected because genetic mapping provides only relative position of the markers to each other (Sourdille *et al*., 2003). The markers identified and validated for rust and LLS resistance, as reported in Khedikar *et al*. (2010) and Sujay *et al*. (2012), were successfully deployed in GAB for improving foliar disease resistance in three popular varieties of India namely TAG 24, JL 24 and ICGV 91114 (Varshney *et al*., 2014a). Several of these improved lines have shown 39%–79% higher pod yield and 25%–89% higher mean haulm yield over original parents in addition to keeping intact early maturity, drought tolerance and other desirable pod features (Janila *et al*., 2016). Several promising lines are under multilocation testing under All India Coordinated Research Project on Groundnut (AICRP‐G), India, for possible varietal release.

Currently available linked SSR markers for foliar disease resistance are not user‐friendly as they need to be genotyped on PAGE which is tedious and time taking. In this study, successful deployment of QTL‐seq approach identified putative candidate genes and development of user‐friendly diagnostic markers for rust and LLS resistance. In this context, the RIL population (TAG 24 × GPBD 4) was used for making bulks with extreme phenotypes for both foliar fungal diseases, that is rust and LLS. This RIL population showed good phenotypic variability for both diseases and was utilized for conducting genetic mapping and QTL analysis resulting in identification of major QTLs for both diseases (Kolekar *et al*., 2016; Sujay *et al*., 2012). The number of samples to be used in pooling was higher than any other previous studies such as ten samples (Das *et al*., 2015 in chickpea; Lu *et al*., 2014 in cucumber) and 15 samples (Singh *et al*., 2016a in pigeonpea; Singh *et al*., 2016 in chickpea). The increased number of samples for pooling provided high accuracy in SNP predictions, and therefore, results obtained in this study are reliable.

As cultivated groundnut is tetraploid crop with two different subgenomes (A and B), and therefore, more sequence data were generated than the other studies conducted in diploid species to achieve optimum genome coverage and read depth. The genome size of A‐genome progenitor (*Arachis duranensis*) and B‐genome progenitor (*Arachis ipaensis*) has been estimated to be 1.1 and 1.4 Gb, respectively (Bertioli *et al*., 2016). In the case of diploid species of medium genome sized crop plants, mere 57–65 million reads were generated (Das *et al*., 2015 in chickpea; Lu *et al*., 2014 in cucumber; Singh *et al*., 2016a in pigeonpea and Singh *et al*., 2016 in chickpea) and successfully achieved higher (>90%) genome coverage. Keeping in mind the large genome size, 365.22–423.76 million reads were generated which helped in successfully achieving 86.57%–86.86% genome coverage and 11.0–11.6 X average read depth for resistant parent (GPBD 4) and different resistant and susceptible bulks. The above generated sequencing data with moderate genome coverage and read depth allowed for detailed sequence analysis. The possible reasons behind moderate genome coverage include sequencing library used, sequencing errors, structural rearrangements or insertions in the query genome, or deletions in the reference genome (Sims *et al*., 2014).

Upon analysing the sequence data generated for resistant and susceptible bulk samples in comparison with the GPBD 4 assembly, genomic region of 3.06 Mb (131.60–134.66) for rust resistance and 2.98 Mb (131.67–134.65) for LLS resistance on the A‐genome, that is A03, were identified with >99% significance (Figures 1, 2, 3). Gowda *et al*. (2002) indicated that *A. cardenasii* (A‐genome) might be source of resistance alleles present in the resistant genotype, GPBD 4. The above results are of immense importance in confirming the source of resistance, that is A‐genome as above‐mentioned studies did not predict the resistance source. In addition, the present study also provides evidence to the current understanding that the resistance alleles have come from the interspecific derivative, ICGV 86855 (CS16), as this genotype has similar alleles for all the four diagnostic markers to GPBD 4. It is important to note that ICGV 86855 was used as one of the resistant parent while developing the resistant variety, GPBD 4.

**Figure 4:**
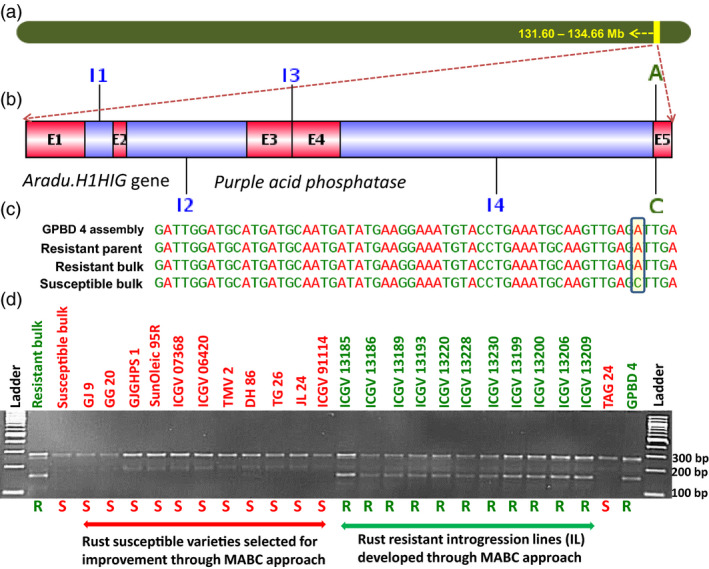
Validation of putative candidate gene‐based marker for rust resistance. (a) Pseudomolecule A03 of *Arachis duranensis* showing genomic region explaining 83.6% PVE for rust resistance, (b) putative candidate gene *Aradu.H1HIG* gene which produces purple acid phosphatase (E1 to E5 refer to exon numbers while I1 to I4 refer to intron numbers), (c) SNP variation in *Aradu.H1HIG* gene and (d) marker validation on a validation set comprising on a set comprising bulks (resistant and susceptible), susceptible genotypes (GJ 9, GJ 20, GJGHPS 1, SunOleic 95R, ICGV 07368, ICGV 06420, TMV 2, DH 86, TG 26, ICGV 91114 and JL 24), both the parents (TAG 24 and GPBD 4).

**Figure 5:**
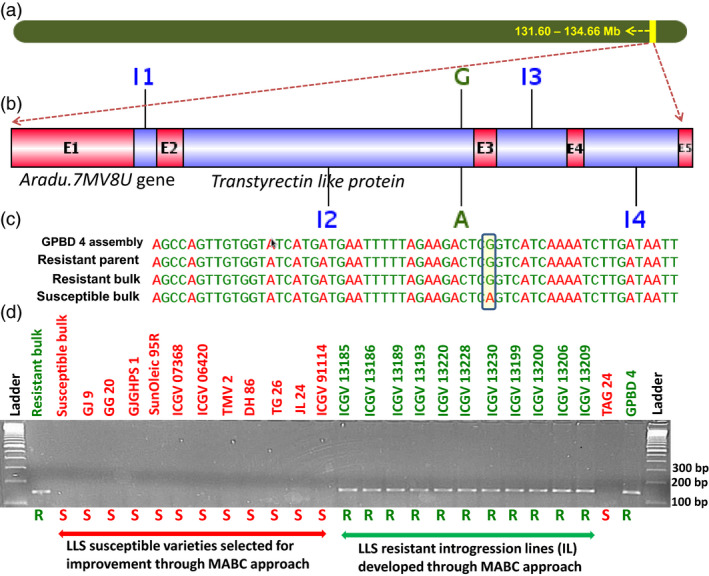
Validation of putative candidate gene‐based marker for late leaf spot resistance. (a) Pseudomolecule A03 of *Arachis duranensis* showing genomic region explaining 83.6% PVE for controlling late leaf spot resistance, (b) putative candidate gene *Aradu.7MV8U* gene which produces transthyrectin‐like protein (E1 to E5 refer to exon numbers while I1 to I4 refer to intron numbers), (c) SNP variation in *Aradu.7MV8U* gene and (d) marker validation on a validation set comprising on a set comprising bulks (resistant and susceptible), susceptible genotypes (GJ 9, GJ 20, GJGHPS 1, SunOleic 95R, ICGV 07368, ICGV 06420, TMV 2, DH 86, TG 26, ICGV 91114 and JL 24), both parents (TAG 24 and GPBD 4) of mapping population and selected introgression lines (four in the genetic background of ICGV 91114, three in JL 24 and four in TAG 24) developed through marker‐assisted backcrossing (MABC) approach.

For rust resistance, a total of 3136 SNPs were identified with the contribution of resistant alleles from the resistant parent GPBD 4 and susceptible alleles from the susceptible parent TAG 24. Total 30 nonsynonymous SNPs affecting 25 putative candidate genes related to plant growth and defence mechanism were identified. Similarly for LLS resistance, 66 SNPs were identified indicating GPBD 4 as the source for resistance alleles and TAG 24 for susceptible alleles. As none of the identified SNP was nonsynonymous in nature, 17 SNPs (14 intronic and three synonymous) representing nine putative candidate genes were targeted for identification of diagnostic markers for LLS resistance. Of the 25 putative candidate genes identified for rust resistance and nine putative genes for LLS resistance, based on the marker validation results in this study, four interesting putative candidate genes were found with their possible role in contributing towards providing genetic resilience against the fungal pathogens. Two putative candidate genes namely *Aradu.PNQ8T* and *Aradu.6U7NW* identified for rust resistance are reported to code for unknown/uncharacterized proteins, and therefore, their further role could not be predicted. One putative candidate gene each for rust, that is *Aradu.H1HIG* (Figure 4), and LLS, that is *Aradu.7MV8U* (Figure 5), are known to code for purple acid phosphatase (PAP) and transthyretin‐like protein, respectively. Interestingly, the *Aradu.7MV8U* gene showed maximum number of effective SNPs (five SNPs) among all putative candidate genes identified in this study. More interestingly, the putative candidate gene *Aradu.7MV8U* was identified for both the fungal diseases, therefore, seems to be very important in providing disease resistance against the fungal diseases.

The phosphatases are well known for their key role in the production, transport and recycling of inorganic phosphorus which not only helps the cellular metabolism and bioenergetics but also play important role in bacterial killing (Kaida *et al*., 2010). The degradation of DNA by PAPs from yellow lupin seeds implies a role in plant growth and repair and in pathogen defence (Antonyuk *et al*., 2014). On the other hand, the putative candidate gene *Aradu.7MV8U* which produces transthyretin‐like protein seems to play important role in plant growth and defence. It is reported that *Arabidopsis thaliana* transthyretin‐like protein (TTL) serves as a potential substrate to BRASSINOSTEROID‐INSENSITIVE 1 (BRI1), a leucine‐rich‐repeat (LRR) receptor kinase that functions as a critical component of a transmembrane BR receptor (Nam and Li, 2004). It is believed that BRI1 becomes activated through hetero‐dimerization with BRI1‐associated receptor kinase 1 (BAK1), a similar LRR receptor kinase, in response to BR signal. As this putative candidate gene has been detected for both the fungal foliar diseases, further study is required to gain insights on their specific role in defence mechanism for both the foliar fungal diseases. More than 80 different mutations in the transthyretin (TTR) gene have been identified in human leading to several diseases (http://www.genecards.org/cgi-bin/carddisp.pl?gene=TTR). For example, one of its variant known as ‘TTR‐52’ produces TTR‐52 protein in *Caenorhabditis elegans*, which facilitates recognition of apoptotic cells (Wang *et al*., 2010). It is important to note that phagocytosis and removal of apoptotic cells are the key process in tissue remodelling, suppression of inflammation and regulation of immune response in humans (Henson *et al*., 2001; Savill *et al*., 2002).

Allele‐specific markers which can be simply scored on agarose gel electrophoresis are the most cost‐effective assays to genotype the breeding population in order to select plants with desired allele. Of the 45 SNPs targeting 34 putative candidate genes, allele‐specific primers were successfully developed for 25 SNPs targeting 25 putative candidate genes. Further, of the 25 SNPs, primers were designed for 20 SNPs for both alleles, while for remaining five SNPs, only one allele could be developed. The possible solution to such a problem is to design allele‐specific primers with an additional base pair mismatch of the third bases close to the SNP site between alleles. Albeit, designing primer for other mismatches to increase primer particularity is a tough for more number allele‐specific markers (Liu *et al*., 2012). Of the 45 primers tested, 36 were amplified and four of these were found polymorphic. Despite designing primers for both the alleles of each SNP, amplification of markers was not observed for both the alleles of a SNP. Nonamplification of few markers may be due to not perfectly complemented to the DNA template (You *et al*., 2008). It was observed that three of these markers amplified resistance allele, while marker ‘GMRQ843’ amplified susceptible allele. It was interesting to note that complementary allele of these markers was found monomorphic which might be due to nondiscrimination between the alleles of a SNP.

QTL analysis using the genotyping (including four new marker loci) and phenotyping data identified 11 QTLs with comparatively higher LOD value and phenotypic variance. It was encouraging to note that newly developed marker ‘GMRQ517’ flanked the QTL region across seasons with GM1536. Four polymorphic markers identified on parental genotypes were further validated on a panel of genotypes containing susceptible genotypes, both the parents of mapping population and selected introgression lines. Three of these markers have shown clear differentiation between resistant and susceptible genotypes for rust resistance, while one diagnostic marker was identified for LLS resistance. It is worth mentioning here that two diagnostic markers for rust resistance can be used in combination (GMRQ517 + GMRQ843) in the segregating population to differentiate the homozygotes and heterozygotes.

In summary, the currently deployed genetic markers from the previous study for selecting resistant plants in the field are not user‐friendly as they require not only skill and technical expertise but also are expensive and not cost‐effective. This study has provided allele‐specific PCR‐based markers for both diseases which are user‐friendly as they can be simply scored on agarose gel electrophoresis. These newly developed markers are cost‐effective and very easy to genotype for developing improved groundnut lines with enhanced resistance to LLS and rust.

## Materials and methods

### Plant materials and construction of bulks

The RIL mapping population TAG 24 × GPBD 4 comprising of 266 individuals was used in this study. The resistant parent, GPBD 4, is derived from the cross KRG 1 × ICGV 86855 (CS 16) and is used as a national check for resistance to both foliar fungal diseases, that is rust and LLS resistance in All India Coordinated Research Project on Groundnut (AICRP‐G) in India. It is important to note that ICGV 86855 (CS 16), an interspecific derivative of *A. cardenasii*, was the resistance source for both diseases in breeding GPBD 4 variety (Gowda *et al*., 2002). In addition to the disease resistance, this variety is popular in the Karnataka state of India because of its good agronomic features such as medium maturity duration, high yield and high pod growth rate with high oil content (Sujay *et al*., 2012). The susceptible parent ‘TAG 24’ of the RIL population is an early maturing popular variety with high harvest index, better partitioning coefficient and tolerance to bud necrosis, but is highly susceptible to rust and LLS diseases (Sujay *et al*., 2012). Extensive phenotyping data for rust and LLS resistance were assembled at the University of Agricultural Sciences, Dharwad, India, for 6 years/seasons (2004–2009). The details on phenotyping were provided by Sujay *et al*. (2012). The above‐mentioned phenotyping data were used for construction of two bulks with extreme phenotypes, that is resistant and susceptible bulks in this study (Figure S1; Table S1).

DNA isolated from 25 RILs with lowest rust disease score was pooled to constitute rust resistance bulk (Rust_Rbulk), while DNA from 25 RILs with highest disease score was pooled to constitute rust susceptible bulk (Rust_Sbulk) (Figure S2). Similarly, resistance (LLS_Rbulk) and susceptible (LLS_Sbulk) bulks were constituted for LLS resistance.

### Construction of sequencing libraries and Illumina sequencing

A total of five samples, that is resistant parent (GPBD 4), resistant bulk for rust (Rust_Rbulk), susceptible bulk for rust (Rust_Sbulk), resistant bulk for LLS (LLS_Rbulk) and susceptible bulk for LLS (LLS_Sbulk), were prepared and used for sequencing on Illumina HiSeq 2500 (Illumina Inc., San Diego, CA, USA). One Illumina library each was prepared for all the five samples using TruSeq DNA Sample Prep kit LT, (set A) FC‐121‐2001. To construct a library, 2 μg DNA from each of these five samples was first sheared using diagenode Bioruptor^®^ NGS (Diogenode, Liege, Belgium) and then was subjected to end repairing and adapter ligation. Realizing the importance of size selection for use in resequencing, 2% agarose gel was used for size separation and selected desired insert size of 500–600 bp. These selected libraries of desired sizes were first purified and then enriched using adaptor compatible PCR primers. To ensure size distribution of libraries, the amplified DNA libraries were also checked on an Agilent Technologies 2100 Bioanalyzer (Agilent Technologies, Palo Alto, CA, USA) using a high‐sensitivity chip. These selected DNA libraries were then used for generating 250 bases pair‐end reads by sequencing on Illumina HiSeq platform with Reagent Kit v2 (500‐cycles).

### Construction of reference‐guided assembly for the resistant parent

After generating the sequence on all five samples, the QTL‐seq pipeline (http://genome-e.ibrc.or.jp/home/bioinformatics-team/mutmap) was used for calculating SNP index. This pipeline was developed at Iwate Biotechnology Research Center, Japan. A reference tetraploid genome assembly was developed using diploid genome assemblies of both the progenitors, that is assemblies for A‐genome (*A. duranensis*) and B‐genome (*A. ipaensis*) (Bertioli *et al*., 2016). After downloading and installing the QTL‐seq pipeline, the cleaned reads of resistant parent (GPBD 4) were first aligned to the above‐mentioned reference tetraploid genome assembly using inbuilt BWA aligner. After aligning sequence reads to both diploid genomes separately, the Coval software was used for postprocessing and filtering of the alignment files (Kosugi *et al*., 2013). The variants were called between resistant parent (GPBD 4) and both diploid reference genomes. These variants were then used to develop reference‐guided assembly of the resistant parent; GPBD 4 (hereafter referred as GPBD 4 assembly) using synthetic tetraploid genome assembly by substituting the bases with confidence variants calls in the genome. After developing GPBD 4 assembly, the reads from rust and LLS resistance (both resistant and susceptible bulks) were then aligned onto GPBD 4 assembly. The variants (SNP index) were then called for all the four bulk samples with GPBD 4 assembly.

### Calculation of SNP index

SNP index for both the set of bulks was calculated by comparing with the GPBD 4 assembly following the formula suggested by Abe *et al*. (2012). SNP index at a position in a pseudomolecule is derived by division of the counts of alternate base with the number of reads aligned. The SNP positions with read depth <7 in both the bulks and SNP index <0.3 in either of the bulks were filtered out. ∆SNP index was then calculated by subtracting SNP index of resistant bulk from SNP index of susceptible bulk. It is important to mention that only those SNPs were selected for ∆SNP index calculation that had homozygous alleles in both bulks, that is resistant as well as susceptible. Further, only those SNP positions considered as the causal SNPs responsible for the trait of interest which passed the criteria of having ∆SNP index = −1. ∆SNP index = −1 indicate that the allele called in resistant bulk was same as that of resistant parent while alternate base in susceptible bulk (Figure S3). As the QTLs for both the resistance traits were found in A03 pseudomolecule, emphasis was given more on the SNP indices calculated for the pseudomolecule A03 for further discovery of candidate genes and marker development.

### Marker–trait association and re‐estimation of QTL effect

Based on the SNP index values of rust and LLS bulks, allele‐specific primers were designed for markers targeting the promising SNPs differentiating the bulks using BatchPrimer3 (You *et al*., 2008). Genotyping for these markers was done following the PCR conditions explained in Varshney *et al*. (2009) and Sujay *et al*. (2012). After PCR amplification, the alleles were scored on 2% agarose gel as present and absent. Initially, all the markers were amplified on both parents (TAG 24 and GPBD 4) of the RIL population. The genotyping data for newly developed polymorphic markers were generated on complete RIL population and were integrated into the linkage group of existing genetic map using JoinMap 4.0 (Van Ooijen, 2006). The Kosambi map function (Kosambi, 1944) with recombination frequency of 0.45 was used achieving the map order for these new markers by keeping the order fixed for earlier marker loci. The genetic map for this linkage group was then redrawn using MapChart for Windows for better visualization (Voorrips, 2002). This genetic information together with phenotyping data was used for conducting QTL analysis using the composite interval mapping model in the software WinQTL cartographer 2.5 (Wang *et al*., 2007). The optimum analysis parameters were set for the analysis such as 1 cM walking speed, 10 cM window size and 5 cM for number of control markers. The QTLs which had LOD values >2.5 were considered as ‘significant’ QTLs.

### Validation of allele‐specific markers

The above‐mentioned four promising markers were validated on a set of 26 samples including 12 introgression lines and a set of ten susceptible parental lines, parents of RIL population and both the bulks to see their utility in GAB. The introgression lines included four ILs (ICGV 13185, ICGV 13186, ICGV 13189 and ICGV 13193) in the genetic background of ICGV 91114, three ILs (ICGV 13120, ICGV 13128 and ICGV 13130) in the genetic background of JL 24 and four ILs (ICGV 13199, ICGV 13200, ICGV 13206 and ICGV 13209) in the genetic background of TAG 24. These introgression lines were developed using marker‐assisted backcrossing (MABC) approach (Varshney *et al*., 2014a) using the then available linked SSR markers (IPAHM103, GM2301, GM1536 and GM2079) for the rust resistance in groundnut identified by Khedikar *et al*. (2010) and Sujay *et al*. (2012). Remaining ten genotypes included nine susceptible genotypes namely TMV 2, GJ 9, GG 20, GJGHPS1, SunOleic 95R, ICGV 07368, DH 86, TAG 24 and TG 26 and one resistant genotype, GPBD 4.

## Author contributions

M.K.P. performed most of the experiments; V.K. and A.C. generated sequence data; A.W.K. and V.G. performed computational analysis; M.K.V. designed primers; R.B. and P.J. contributed genetic material; M.K.P., V.K.S., B.G. and R.K.V. analysed and interpreted the results; M.K.V. and Y.S. performed validation of candidate SNPs/markers; M.K.P. prepared the first draft; M.K.P. and V.K.S. prepared improved manuscript; M.K.P. and R.K.V. conceived, designed and supervised the study and finalized the manuscript.

## Conflict of interest

The author(s) declare that they have no competing interests.

## Supporting information


**Figure S1** Phenotypic variability among the RILs selected for development of resistance and susceptible pools for rust and late leaf spot diseases.

**Figure S2** QTL‐seq approach used for trait mapping in groundnut for rust and late leaf spot resistance.

**Figure S3** Alignment, SNP identification and calculation of SNP index for rust and late leaf spot resistance.

**Figure S4** Sequencing depth of the resistant parent GPBD 4.

**Figure S5** SNP index plots for 20 pseudomolecules of rust resistant bulk with the resistant parent.

**Figure S6** SNP index plots for 20 pseudomolecules of rust susceptible bulk with resistant parent.

**Figure S7** The Δ(SNP index) plot obtained by subtraction of rust resistant pool SNP index from rust susceptible pool SNP index.

**Figure S8** SNP index plots for 20 pseudomolecules of LLS resistant bulk with the resistant parent.

**Figure S9** SNP index plots for 20 pseudomolecules of LLS susceptible bulk with resistant parent.

**Figure S10** The Δ(SNP index) plot obtained by subtraction of LLS resistant pool SNP index from rust susceptible bulk SNP index.

**Figure S11** Integration of newly identified diagnostic markers on genetic map and estimation of QTL effects.

**Figure S12**Validation of four identified diagnostic markers in a set of germplasm for rust and late leaf spot resistance.

**Table S1** Details on the recombinant inbred lines (RILs) selected for construction of resistant and susceptible bulks.

**Table S2** Details on whole‐genome resequencing data generated on parental genotypes and bulked samples using Illumina HiSeq 2500.

**Table S3** Pseudomolecule‐wise SNPs distribution between resistant and susceptible bulks for rust and late leaf spot resistance.

**Table S4** Identification of SNPs between resistant and susceptible bulks using QTL‐seq approach for rust resistance.

**Table S5** Identification of SNPs between resistant and susceptible bulks using QTL‐seq approach for late leaf spot resistance.

**Table S6** List of allele‐specific primers developed for rust and late leaf spot resistance.

**Table S7** Associated markers identified on A03 for rust and late leaf spot resistance using single marker analysis (SMA).
