# Supplementary material for: QTL‐seq approach identified genomic regions and diagnostic markers for rust and late leaf spot resistance in groundnut ( Arachis hypogaea L.)
Source: Plant Biotechnol J. 2017 Feb 7;15(8):927–41. doi: 10.1111/pbi.12686 (PMC5506652; doi:10.1111/pbi.12686)
Supplement: Supplementary file 1 — Figure S1 Phenotypic variability among the RILs selected for development of resistance and susceptible pools for rust and late leaf spot diseases. [file PBI-15-927-s001.pptx]

## Slide 1
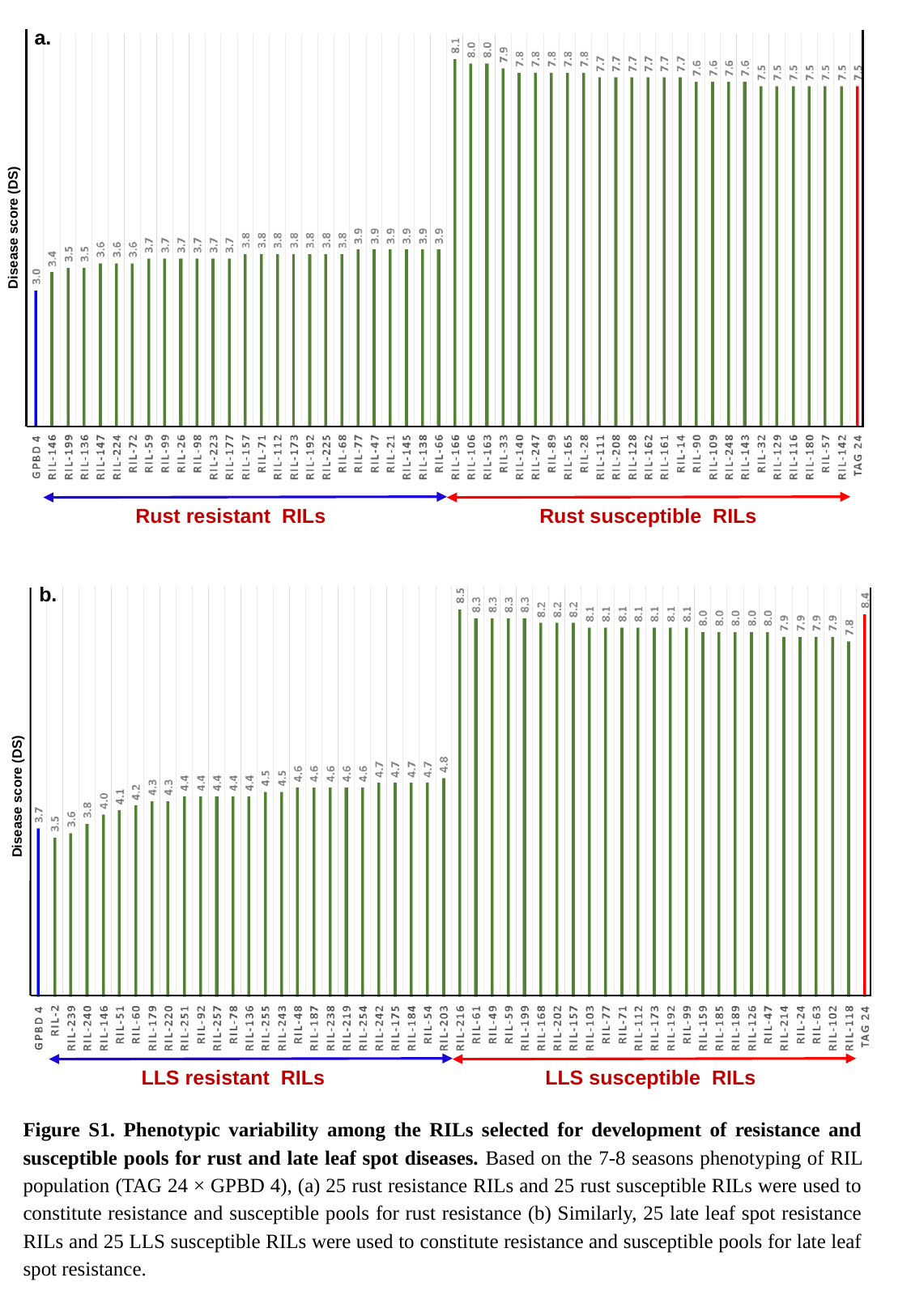

a.
Rust resistant RILs
Rust susceptible RILs
Disease score (DS)
 b.
Disease score (DS)
LLS resistant RILs
LLS susceptible RILs
Figure S1. Phenotypic variability among the RILs selected for development of resistance and susceptible pools for rust and late leaf spot diseases. Based on the 7-8 seasons phenotyping of RIL population (TAG 24 × GPBD 4), (a) 25 rust resistance RILs and 25 rust susceptible RILs were used to constitute resistance and susceptible pools for rust resistance (b) Similarly, 25 late leaf spot resistance RILs and 25 LLS susceptible RILs were used to constitute resistance and susceptible pools for late leaf spot resistance.
