# Supplementary material for: QTL‐seq approach identified genomic regions and diagnostic markers for rust and late leaf spot resistance in groundnut ( Arachis hypogaea L.)
Source: Plant Biotechnol J. 2017 Feb 7;15(8):927–41. doi: 10.1111/pbi.12686 (PMC5506652; doi:10.1111/pbi.12686)
Supplement: Supplementary file 2 — Figure S2 QTL‐seq approach used for trait mapping in groundnut for rust and late leaf spot resistance. [file PBI-15-927-s017.pptx]

## Slide 1
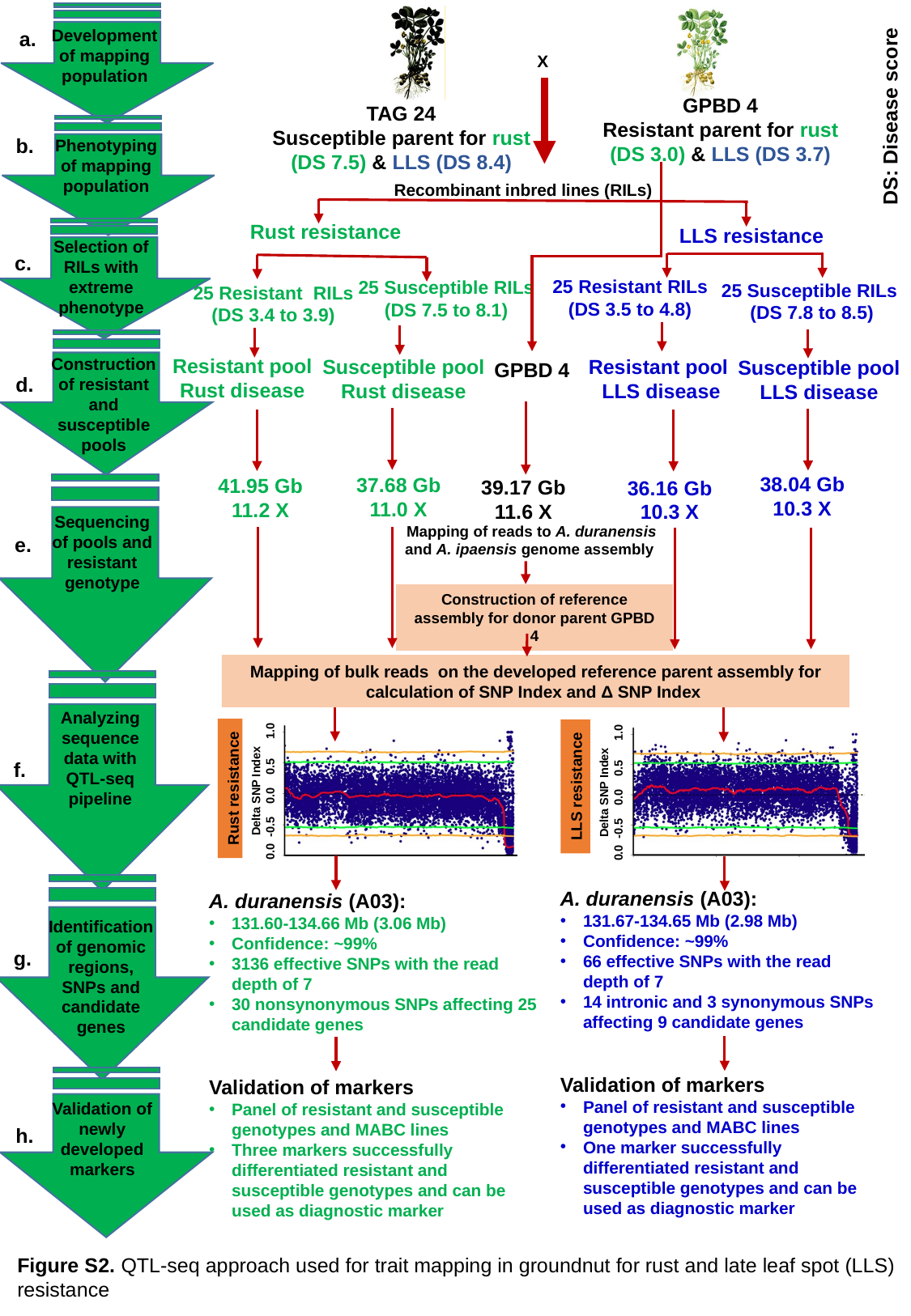

Development of mapping population
 a.
X
TAG 24
Susceptible parent for rust (DS 7.5) & LLS (DS 8.4)
Recombinant inbred lines (RILs)
Rust resistance
LLS resistance
25 Resistant RILs (DS 3.5 to 4.8)
25 Susceptible RILs (DS 7.5 to 8.1)
25 Susceptible RILs
(DS 7.8 to 8.5)
Resistant pool
Rust disease
Resistant pool
LLS disease
Susceptible pool
Rust disease
Susceptible pool
LLS disease
GPBD 4
38.04 Gb
10.3 X
37.68 Gb
11.0 X
41.95 Gb
11.2 X
39.17 Gb
11.6 X
36.16 Gb
10.3 X
Mapping of reads to A. duranensis and A. ipaensis genome assembly
Construction of reference assembly for donor parent GPBD 4
Mapping of bulk reads on the developed reference parent assembly for calculation of SNP Index and Δ SNP Index
LLS resistance
Rust resistance
A. duranensis (A03):
131.67-134.65 Mb (2.98 Mb)
Confidence: ~99%
66 effective SNPs with the read depth of 7
14 intronic and 3 synonymous SNPs affecting 9 candidate genes
Validation of markers
Panel of resistant and susceptible genotypes and MABC lines
One marker successfully differentiated resistant and susceptible genotypes and can be used as diagnostic marker
A. duranensis (A03):
131.60-134.66 Mb (3.06 Mb)
Confidence: ~99%
3136 effective SNPs with the read depth of 7
30 nonsynonymous SNPs affecting 25 candidate genes
Validation of markers
Panel of resistant and susceptible genotypes and MABC lines
Three markers successfully differentiated resistant and susceptible genotypes and can be used as diagnostic marker
GPBD 4
Resistant parent for rust (DS 3.0) & LLS (DS 3.7)
DS: Disease score
 b.
Phenotyping of mapping population
Selection of RILs with extreme phenotype
 c.
25 Resistant RILs (DS 3.4 to 3.9)
Construction of resistant and susceptible pools
 d.
Sequencing of pools and resistant genotype
 e.
Analyzing sequence data with QTL-seq pipeline
 f.
Delta SNP Index
0.0 -0.5 0.0 0.5 1.0
Delta SNP Index
0.0 -0.5 0.0 0.5 1.0
Identification of genomic regions, SNPs and candidate genes
 g.
Validation of newly developed markers
 h.
Figure S2. QTL-seq approach used for trait mapping in groundnut for rust and late leaf spot (LLS) resistance
