# Supplementary material for: QTL‐seq approach identified genomic regions and diagnostic markers for rust and late leaf spot resistance in groundnut ( Arachis hypogaea L.)
Source: Plant Biotechnol J. 2017 Feb 7;15(8):927–41. doi: 10.1111/pbi.12686 (PMC5506652; doi:10.1111/pbi.12686)
Supplement: Supplementary file 3 — Figure S3 Alignment, SNP identification and calculation of SNP index for rust and late leaf spot resistance. [file PBI-15-927-s016.pptx]

## Slide 1
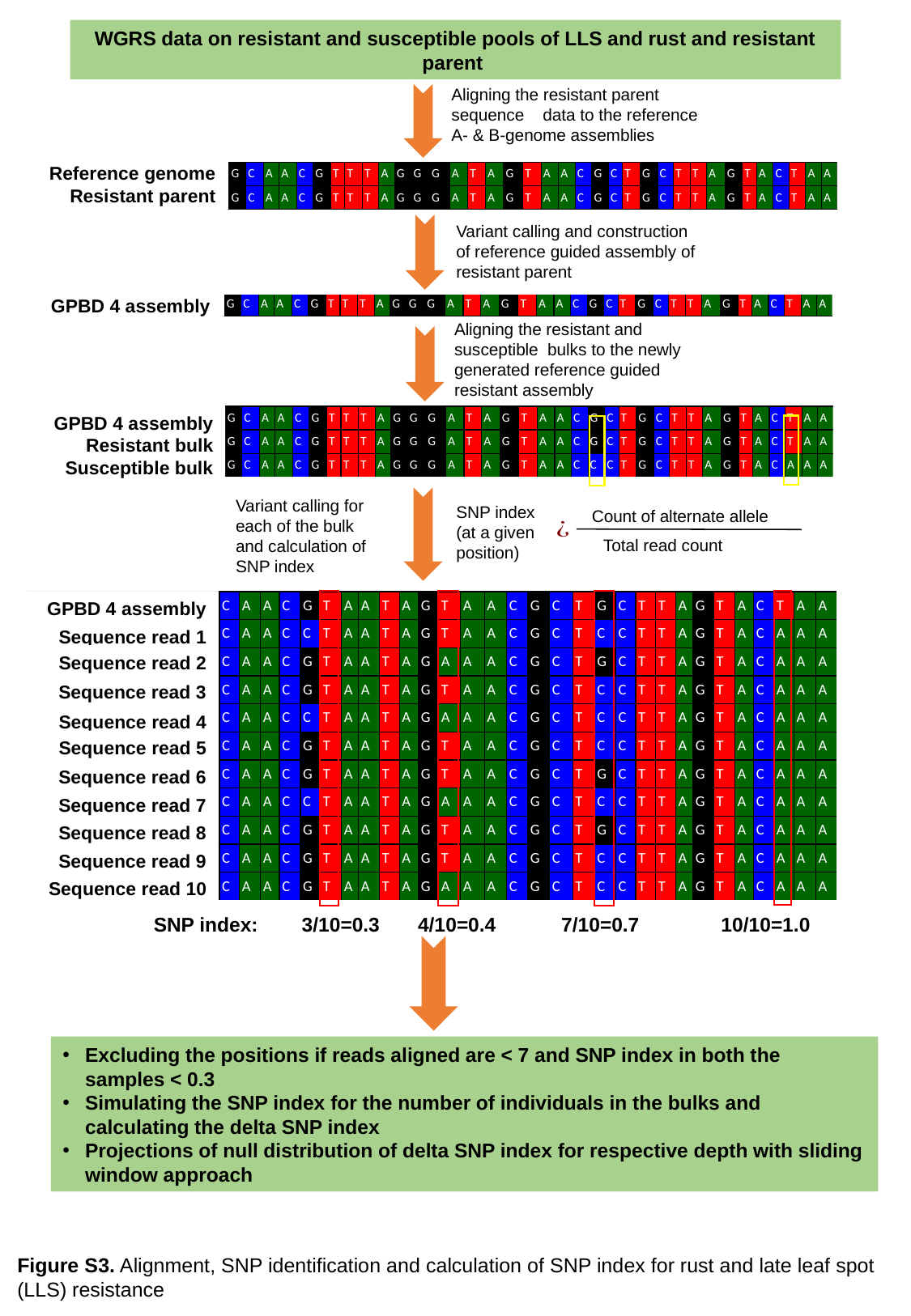

WGRS data on resistant and susceptible pools of LLS and rust and resistant parent
Aligning the resistant parent sequence data to the reference A- & B-genome assemblies
Reference genome Resistant parent
Variant calling and construction of reference guided assembly of resistant parent
GPBD 4 assembly
Aligning the resistant and susceptible bulks to the newly generated reference guided resistant assembly
GPBD 4 assembly
Resistant bulk
Susceptible bulk
Variant calling for each of the bulk and calculation of SNP index
SNP index
(at a given position)
Count of alternate allele
Total read count
SNP index: 3/10=0.3 4/10=0.4 7/10=0.7 10/10=1.0
GPBD 4 assembly
Sequence read 1
Sequence read 2
Sequence read 3
Sequence read 4
Sequence read 5
Sequence read 6
Sequence read 7
Sequence read 8
Sequence read 9
Sequence read 10
Excluding the positions if reads aligned are < 7 and SNP index in both the samples < 0.3
Simulating the SNP index for the number of individuals in the bulks and calculating the delta SNP index
Projections of null distribution of delta SNP index for respective depth with sliding window approach
Figure S3. Alignment, SNP identification and calculation of SNP index for rust and late leaf spot (LLS) resistance
