# Supplementary material for: QTL‐seq approach identified genomic regions and diagnostic markers for rust and late leaf spot resistance in groundnut ( Arachis hypogaea L.)
Source: Plant Biotechnol J. 2017 Feb 7;15(8):927–41. doi: 10.1111/pbi.12686 (PMC5506652; doi:10.1111/pbi.12686)
Supplement: Supplementary file 4 — Figure S4 Sequencing depth of the resistant parent GPBD 4. [file PBI-15-927-s018.pptx]

## Slide 1
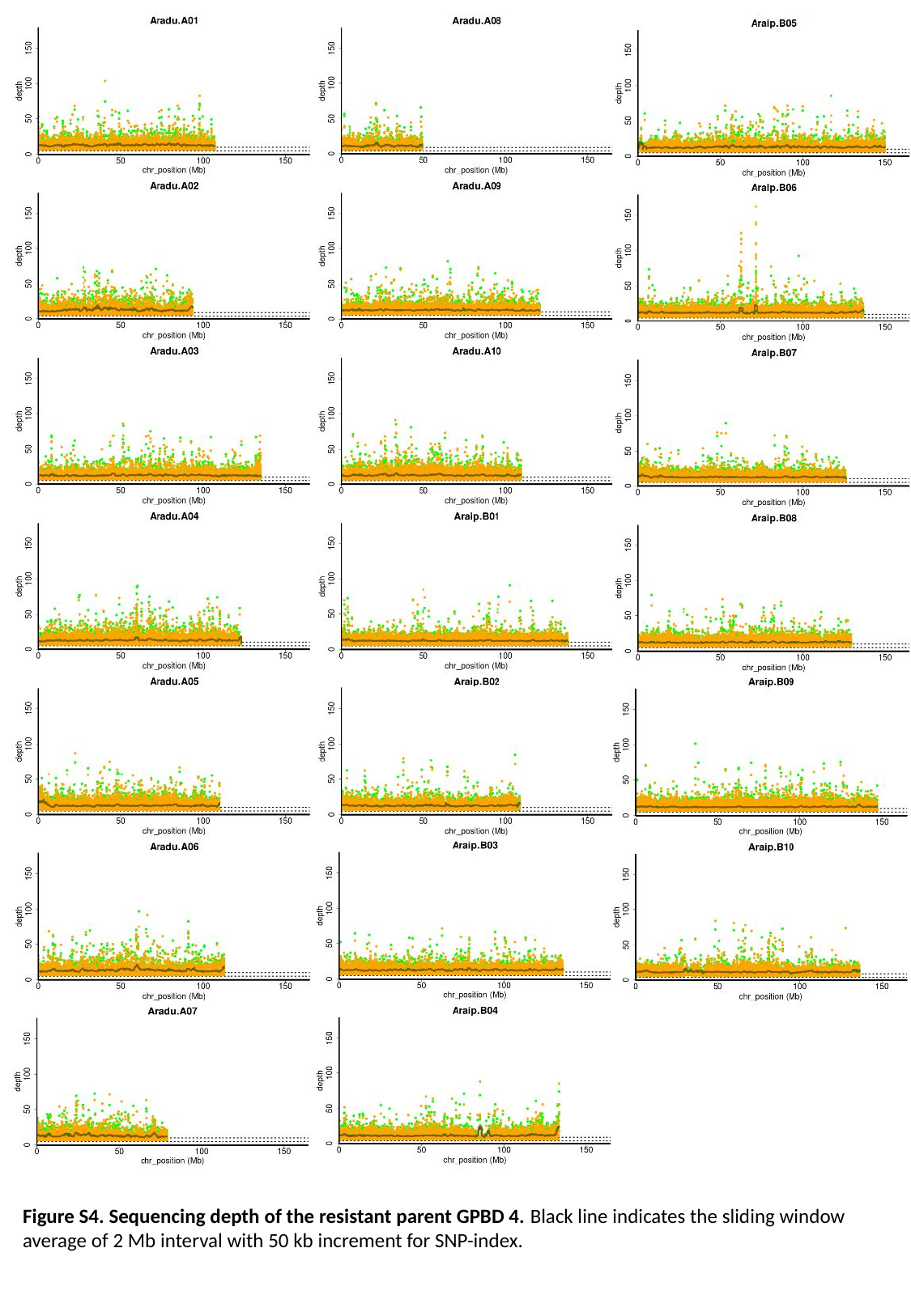

Figure S4. Sequencing depth of the resistant parent GPBD 4. Black line indicates the sliding window average of 2 Mb interval with 50 kb increment for SNP-index.
