# Supplementary material for: QTL‐seq approach identified genomic regions and diagnostic markers for rust and late leaf spot resistance in groundnut ( Arachis hypogaea L.)
Source: Plant Biotechnol J. 2017 Feb 7;15(8):927–41. doi: 10.1111/pbi.12686 (PMC5506652; doi:10.1111/pbi.12686)
Supplement: Supplementary file 5 — Figure S5 SNP index plots for 20 pseudomolecules of rust resistant bulk with the resistant parent. [file PBI-15-927-s019.pptx]

## Slide 1
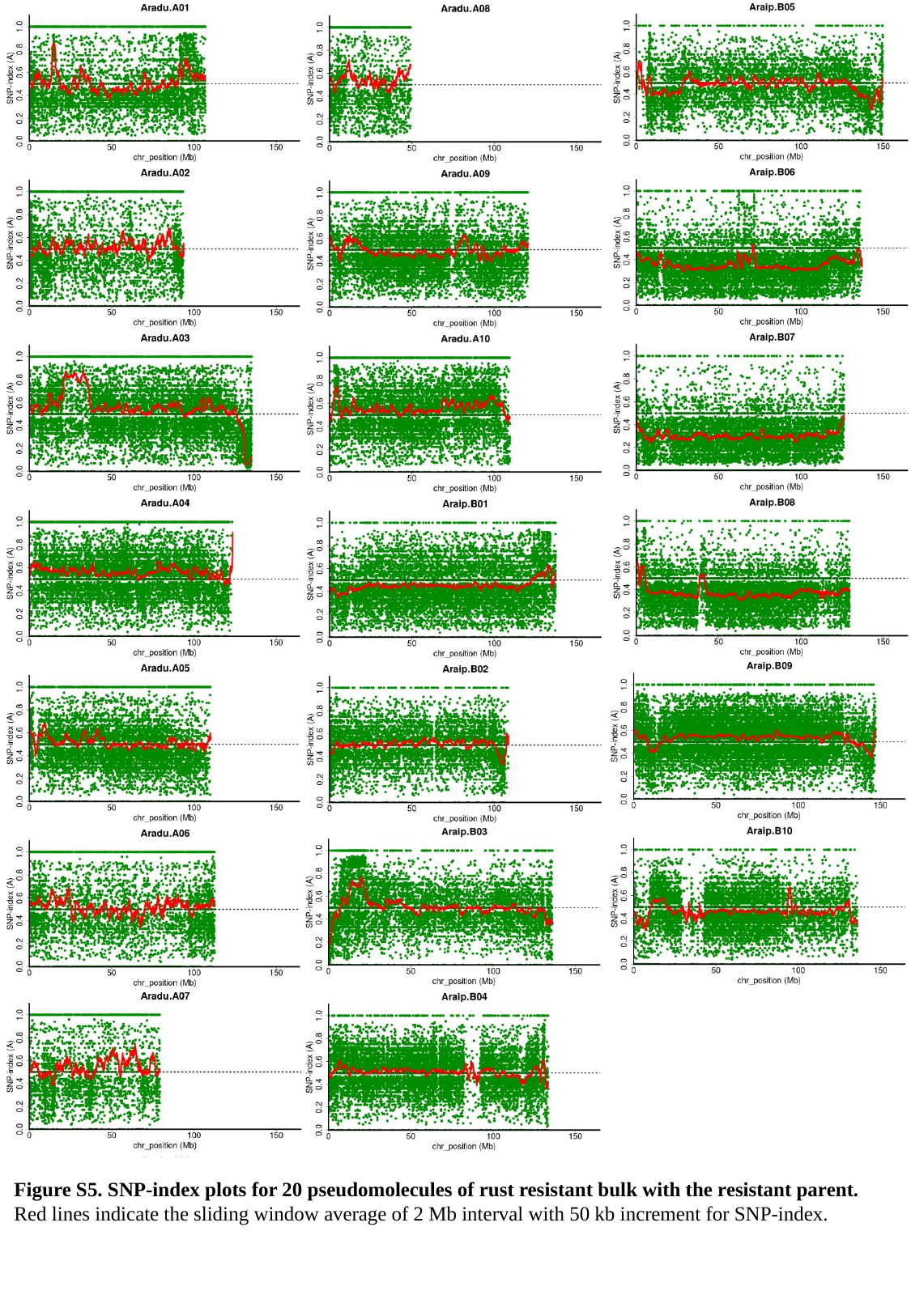

Figure S5. SNP-index plots for 20 pseudomolecules of rust resistant bulk with the resistant parent. Red lines indicate the sliding window average of 2 Mb interval with 50 kb increment for SNP-index.
