# Supplementary material for: QTL‐seq approach identified genomic regions and diagnostic markers for rust and late leaf spot resistance in groundnut ( Arachis hypogaea L.)
Source: Plant Biotechnol J. 2017 Feb 7;15(8):927–41. doi: 10.1111/pbi.12686 (PMC5506652; doi:10.1111/pbi.12686)
Supplement: Supplementary file 10 — Figure S10 The Δ(SNP index) plot obtained by subtraction of LLS resistant pool SNP index from rust susceptible bulk SNP index. [file PBI-15-927-s006.pptx]

## Slide 1
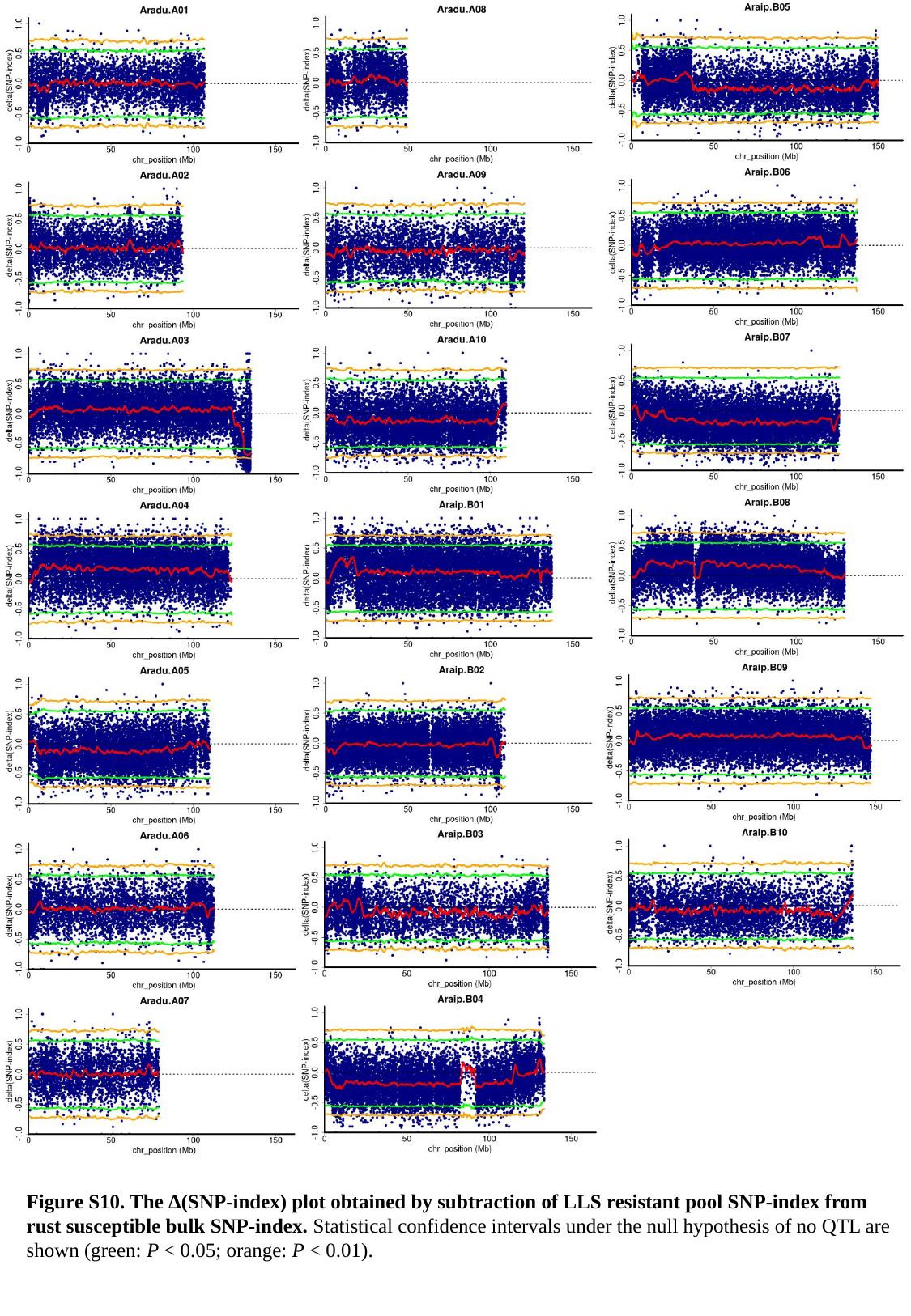

Figure S10. The Δ(SNP-index) plot obtained by subtraction of LLS resistant pool SNP-index from rust susceptible bulk SNP-index. Statistical confidence intervals under the null hypothesis of no QTL are shown (green: P < 0.05; orange: P < 0.01).
