# Supplementary material for: QTL‐seq approach identified genomic regions and diagnostic markers for rust and late leaf spot resistance in groundnut ( Arachis hypogaea L.)
Source: Plant Biotechnol J. 2017 Feb 7;15(8):927–41. doi: 10.1111/pbi.12686 (PMC5506652; doi:10.1111/pbi.12686)
Supplement: Supplementary file 11 — Figure S11 Integration of newly identified diagnostic markers on genetic map and estimation of QTL effects. [file PBI-15-927-s007.pptx]

## Slide 1
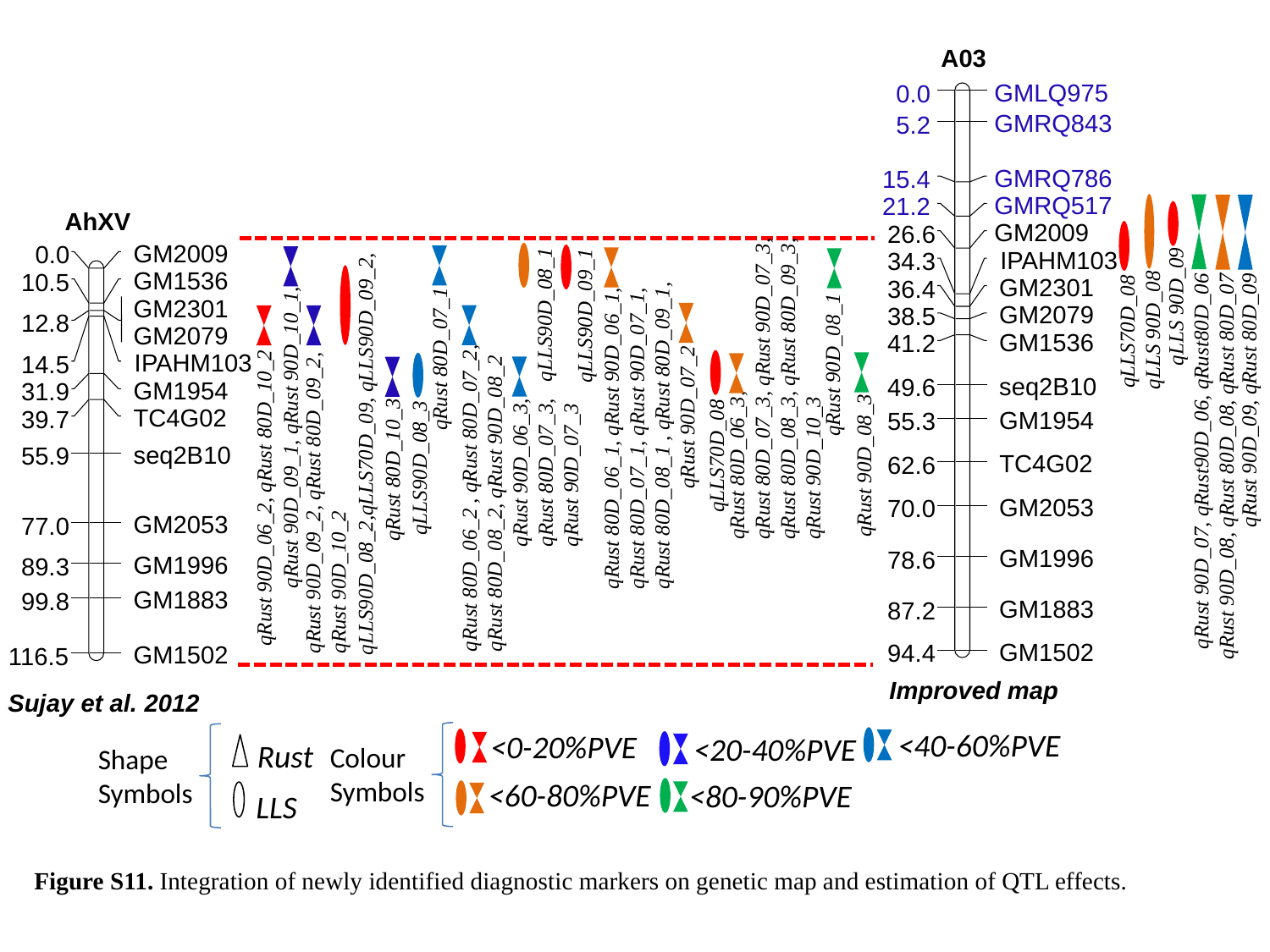

A03
GMLQ975
0.0
GMRQ843
5.2
GMRQ786
15.4
GMRQ517
21.2
GM2009
26.6
IPAHM103
34.3
GM2301
36.4
GM2079
38.5
GM1536
41.2
seq2B10
49.6
GM1954
55.3
TC4G02
62.6
GM2053
70.0
GM1996
78.6
GM1883
87.2
GM1502
94.4
qLLS 90D_09
qLLS 90D_08
qLLS70D_08
qRust 90D_09, qRust 80D_09
qRust 90D_07, qRust90D_06, qRust80D_06
qRust 90D_08, qRust 80D_08, qRust 80D_07
AhXV
GM2009
0.0
GM1536
10.5
GM2301
12.8
GM2079
IPAHM103
14.5
GM1954
31.9
TC4G02
39.7
seq2B10
55.9
GM2053
77.0
GM1996
89.3
GM1883
99.8
GM1502
116.5
qRust 80D_06_3,
qRust 80D_07_3, qRust 90D_07_3,
qRust 80D_08_3, qRust 80D_09_3,
qRust 90D_10_3
qLLS90D_08_1
qLLS90D_09_1
qLLS90D_08_2,qLLS70D_09, qLLS90D_09_2,
qRust 80D_07_1
qRust 90D_09_1, qRust 90D_10_1,
qRust 80D_06_1, qRust 90D_06_1,
qRust 80D_07_1, qRust 90D_07_1,
qRust 80D_08_1 , qRust 80D_09_1,
qRust 90D_08_1
qRust 90D_07_2
qRust 80D_06_2 , qRust 80D_07_2, qRust 80D_08_2, qRust 90D_08_2
qRust 90D_09_2, qRust 80D_09_2,
qRust 90D_10_2
qRust 90D_06_2, qRust 80D_10_2
qRust 90D_08_3
qLLS90D_08_3
qRust 90D_06_3,
qRust 80D_07_3,
qRust 90D_07_3
qRust 80D_10_3
qLLS70D_08
Improved map
Sujay et al. 2012
<40-60%PVE
<0-20%PVE
<20-40%PVE
<60-80%PVE
<80-90%PVE
Colour
Symbols
Rust
LLS
Shape
Symbols
Figure S11. Integration of newly identified diagnostic markers on genetic map and estimation of QTL effects.
