# Supplementary material for: QTL‐seq approach identified genomic regions and diagnostic markers for rust and late leaf spot resistance in groundnut ( Arachis hypogaea L.)
Source: Plant Biotechnol J. 2017 Feb 7;15(8):927–41. doi: 10.1111/pbi.12686 (PMC5506652; doi:10.1111/pbi.12686)
Supplement: Supplementary file 12 — Figure S12Validation of four identified diagnostic markers in a set of germplasm for rust and late leaf spot resistance. [file PBI-15-927-s008.pptx]

## Slide 1
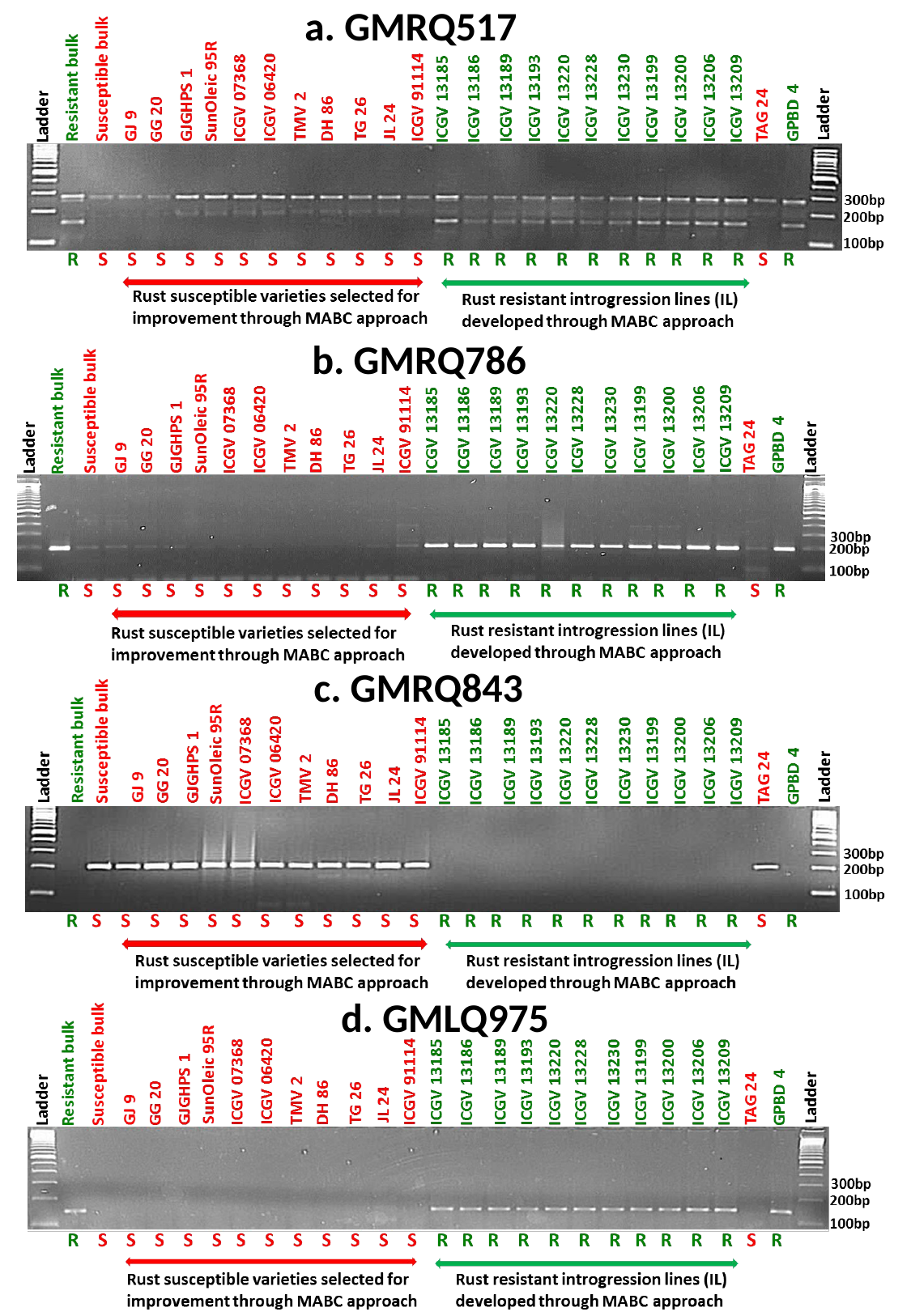

a. GMRQ517
b. GMRQ786
 c. GMRQ843
d. GMLQ975

## Slide 2
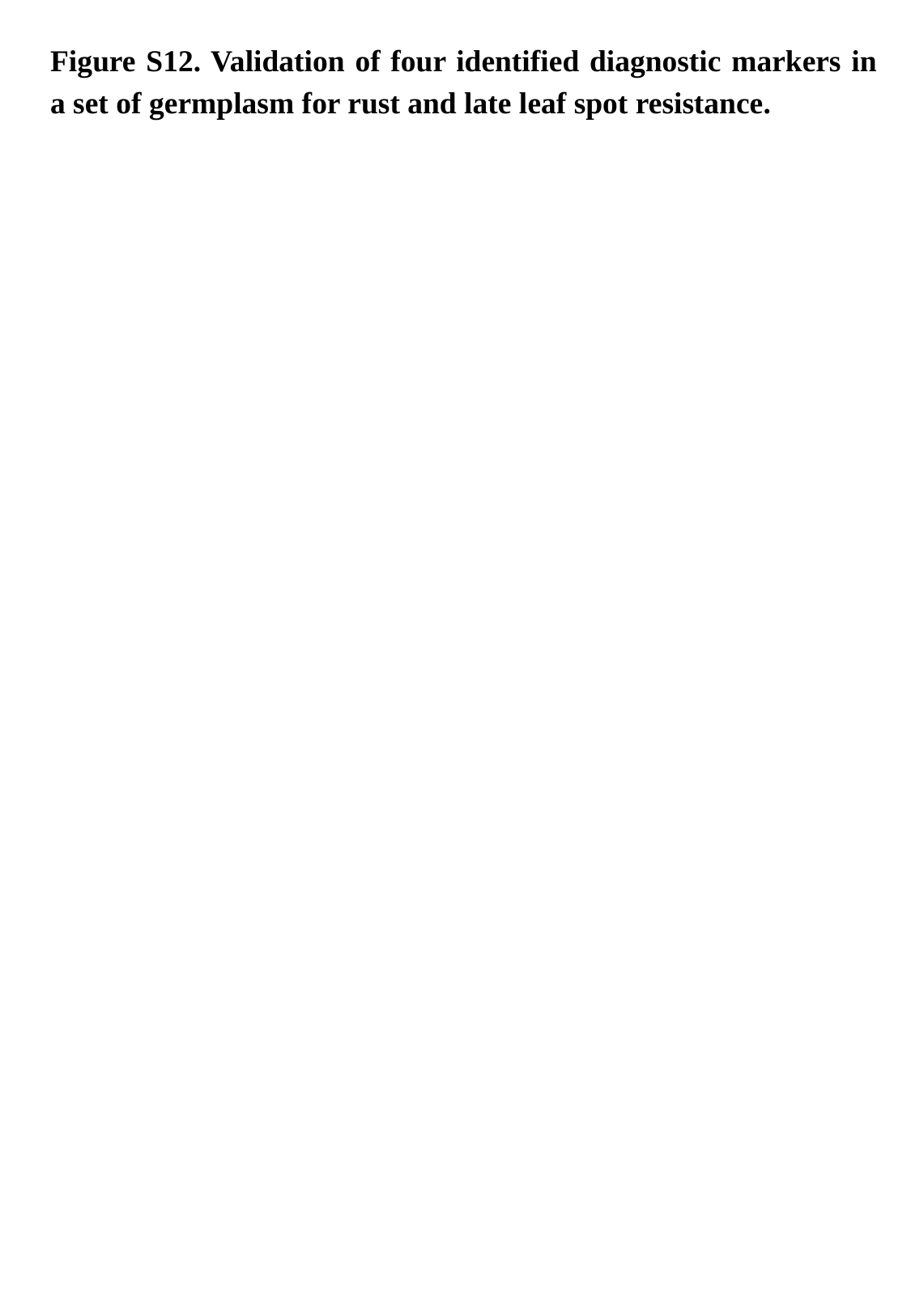

Figure S12. Validation of four identified diagnostic markers in a set of germplasm for rust and late leaf spot resistance.
